# Supplementary material for: Countries’ progress towards Global Health Security (GHS) increased health systems resilience during the Coronavirus Disease-19 (COVID-19) pandemic: A difference-in-difference study of 191 countries
Source: PLOS Glob Public Health. 2025 Jan 7;5(1):e0004051. doi: 10.1371/journal.pgph.0004051 (PMC11706378; doi:10.1371/journal.pgph.0004051)
Supplement: S1 Text — (DOCX) [file pgph.0004051.s002.docx]

**S1 Text. Description of the Dataset**

Our dataset included 18,124 observations spanning 191 countries from 2015 to 2022. Slightly more observations took place in 2020 and 2021 (2,267 observations each) compared with the 2022 (2,255 observations) due to a small number of countries missing data on BCG vaccinations that year. A total of 6,789 (37%) observations took place after the COVID-19 pandemic began in 2020 as compared with 11,335 (63%) which occurred prior to the pandemic.
