# Supplementary material for: Countries’ progress towards Global Health Security (GHS) increased health systems resilience during the Coronavirus Disease-19 (COVID-19) pandemic: A difference-in-difference study of 191 countries
Source: PLOS Glob Public Health. 2025 Jan 7;5(1):e0004051. doi: 10.1371/journal.pgph.0004051 (PMC11706378; doi:10.1371/journal.pgph.0004051)
Supplement: S1 Table — (DOCX) [file pgph.0004051.s003.docx]

**S1 Table. GHSI Categories and Indicators.**

| **Category** | **Indicator** |
| --- | --- |
| 1. Prevention of the Emergence or Release of Pathogens | 1.1 Antimicrobial resistance |
|  | 1.2 Zoonotic disease |
|  | 1.3 Biosecurity |
|  | 1.4 Biosafety |
|  | 1.5 Dual-use research and culture of responsible science |
|  | 1.6 Immunization [*removed from the index for this study*] |
| 2. Early Detection and Reporting Epidemics of Potential International Concern | 2.1 Laboratory systems strength and quality |
|  | 2.2 Laboratory supply chains |
|  | 2.3 Real-time surveillance and reporting |
|  | 2.4 Surveillance data accessibility and transparency |
|  | 2.5 Case-based investigation |
|  | 2.6 Epidemiology workforce |
| 3. Rapid Response to and Mitigation of the Spread of an Epidemic | 3.1 Emergency preparedness and response planning |
|  | 3.2 Exercising response plans |
|  | 3.3 Emergency response operation |
|  | 3.4 Linking public health and security authorities |
|  | 3.5 Risk communication |
|  | 3.6 Access to communications infrastructure |
|  | 3.7 Trade and travel restrictions |
| 4. Sufficient and Robust Health System to Treat the Sick and Protect Health Workers | 4.1 Health capacity in clinics, hospitals, and community care centers |
|  | 4.2 Supply chain for health system and healthcare workers |
|  | 4.3 Medical countermeasures and personnel deployment |
|  | 4.4 Healthcare access |
|  | 4.5 Communications with healthcare workers during a public health emergency |
|  | 4.6 Infection control practices |
|  | 4.7 Capacity to test and approve new medical countermeasures |
| 5. Commitments to Improving National Capacity, Financing Plans to Address Gaps, and Adhering to Global Norms | 5.1 International Health Regulations (IHR) reporting compliance and disaster risk reduction |
|  | 5.2 Cross-border agreements on public and animal health emergency response |
|  | 5.3 International commitments |
|  | 5.4 Joint External Evaluation (JEE) and Performance of Veterinary Services (PVS) Pathway |
|  | 5.5 Financing |
|  | 5.6 Commitment to sharing of genetic and biological data and specimens |
| 6. Overall Risk Environment and Country Vulnerability to Biological Threats | 6.1 Political and security risk |
|  | 6.2 Socio-economic resilience |
|  | 6.3 Infrastructure Adequacy |
|  | 6.4 Environmental Risks |
|  | 6.5 Public health vulnerabilities |
