# Supplementary material for: Countries’ progress towards Global Health Security (GHS) increased health systems resilience during the Coronavirus Disease-19 (COVID-19) pandemic: A difference-in-difference study of 191 countries
Source: PLOS Glob Public Health. 2025 Jan 7;5(1):e0004051. doi: 10.1371/journal.pgph.0004051 (PMC11706378; doi:10.1371/journal.pgph.0004051)
Supplement: S2 Table — (DOCX) [file pgph.0004051.s004.docx]

**S2 Table. GHSI Countries grouped by recomputed GHSI scores.**

| Countries with Overall GHSI$\geq$57 (N=22) | Armenia, Australia, Belgium, Bulgaria, Canada, Denmark, Finland, France, Germany, Japan, Latvia, Netherlands, Norway, Portugal, Republic of Korea, Slovenia, Spain, Sweden, Switzerland, Thailand, United Kingdom, United States. |
| --- | --- |
| Countries with Overall GHSI<57 (N=169) | Afghanistan, Albania, Algeria, Andorra, Angola, Antigua and Barbuda, Argentina, Austria, Azerbaijan, Bahamas, Bahrain, Bangladesh, Barbados, Belarus, Belize, Benin, Bhutan, Bolivia (Plurinational State of), Bosnia and Herzegovina, Botswana, Brazil, Brunei Darussalam, Burkina Faso, Burundi, Cabo Verde, Cambodia, Cameroon, Central African Republic, Chad, Chile, China, Colombia, Comoros, Congo, Costa Rica, Code d’Ivoire, Croatia, Cuba, Cyprus, Czechia, Democratic Republic of the Congo, Djibouti, Dominica, Dominican Republic, Ecuador, Egypt, El Salvador, Equatorial Guinea, Eritrea, Estonia, Eswatini, Ethiopia, Fiji, Gabon, Gambia, Georgia, Ghana, Greece, Grenada, Guatemala, Guinea, Guinea-Bissau, Guyana, Haiti, Honduras, Hungary, Iceland, India, Indonesia, Iran (Islamic Republic of), Iraq, Ireland, Israel, Italy, Jamaica, Jordan, Kazakhstan, Kenya, Kiribati, Kuwait, Kyrgyzstan, Lao People’s Democratic Republic, Lebanon, Lesotho, Liberia, Libya, Lithuania, Luxembourg, Madagascar, Malawi, Malaysia, Maldives, Mali, Malta, Marshall Islands, Mauritania, Mauritius, Mexico, Micronesia (Federated States of), Monaco, Mongolia, Montenegro, Morocco, Mozambique, Myanmar, Namibia, Nauru, Nepal, New Zealand, Nicaragua, Niger, Nigeria, Oman, Pakistan, Palau, Panama, Papua New Guinea, Paraguay, Peru, Philippines, Poland, Qatar, Republic of Moldova, Republic of North Macedonia, Romania, Russian Federation, Rwanda, Saint Kitts and Nevis, Saint Lucia, Saint Vincent and the Grenadines, Samoa, San Marino, Sao Tome and Principe, Saudi Arabia, Senegal, Serbia, Seychelles, Sierra Leone, Singapore, Slovakia, Solomon Islands, Somalia, South Africa, South Sudan, Sri Lanka, Sudan, Suriname, Syrian Arab Republic, Tajikistan, Timor-Leste, Togo, Tonga, Trinidad and Tobago, Tunisia, Türkiye, Turkmenistan, Tuvalu, Uganda, Ukraine, United Arab Emirates, United Republic of Tanzania, Uruguay, Uzbekistan, Vanuatu, Venezuela (Bolivarian Republic of), Viet Nam, Yemen, Zambia, Zimbabwe. |
