# Supplementary material for: Countries’ progress towards Global Health Security (GHS) increased health systems resilience during the Coronavirus Disease-19 (COVID-19) pandemic: A difference-in-difference study of 191 countries
Source: PLOS Glob Public Health. 2025 Jan 7;5(1):e0004051. doi: 10.1371/journal.pgph.0004051 (PMC11706378; doi:10.1371/journal.pgph.0004051)
Supplement: S3 Table — (DOCX) [file pgph.0004051.s005.docx]

**S3 Table. Difference-in-difference model results for overall GHSI and GHSI categories by cutoff values (2020-2022).**

| **GHSI Category** | **Cutoff Value** | **Average DiD effect size (2020-2022)** | **95% Confidence Interval** | ***p-value* for parallel trend** |
| --- | --- | --- | --- | --- |
| Overall GHSI Score (2019) | 30 | 0.24 | -0.32 - 0.804 | 0.00 |
|  | 35 | -0.73 | -1.26 - -0.21 | 0.18 |
|  | 40 | -0.10 | -0.64 - 0.432 | 0.41 |
|  | 45 | -0.50 | -1.05 - 0.042 | 0.51 |
|  | 50 | 0.62 | -0.06 - 1.303 | 0.00 |
|  | 55 | 1.40 | 0.765 - 2.033 | 0.03 |
|  | 60 | 1.04 | 0.581 - 1.497 | 0.11 |
|  | 65 | 0.99 | 0.372 - 1.606 | 0.11 |
|  | 70 | 1.05 | -0.06 - 2.168 | 0.04 |
|  | 75 | 3.14 | 0.611 - 5.674 | 0.05 |
| 1. Prevention of the Emergence or Release of Pathogens | 15 | -0.49 | -0.97 - -0.01 | 0.08 |
|  | 20 | 0.41 | -0.74 - 1.57 | 0.16 |
|  | 25 | -0.17 | -1.13 - 0.78 | 0.02 |
|  | 30 | 0.31 | -0.30 - 0.919 | 0.16 |
|  | 35 | 0.25 | -0.42 - 0.932 | 0.06 |
|  | 40 | 0.56 | -0.11 - 1.239 | 0.43 |
|  | 45 | 0.57 | 0.00 - 1.148 | 0.05 |
|  | 50 | 1.09 | 0.612 - 1.573 | 0.04 |
|  | 55 | 1.09 | 0.528 - 1.656 | 0.01 |
|  | 60 | 1.11 | 0.517 - 1.708 | 0.00 |
|  | 65 | 1.42 | 0.727 - 2.109 | 0.00 |
|  | 70 | 1.49 | 0.064 - 2.914 | 0.75 |
|  | 75 | 1.49 | 0.100 - 2.878 | 0.75 |
| 2. Early Detection and Reporting Epidemics of Potential International Concern | 20 | -0.44 | -0.93 - 0.049 | 0.02 |
|  | 25 | -0.44 | -0.91 - 0.021 | 0.04 |
|  | 30 | -0.58 | -1.07 - -0.10 | 0.01 |
|  | 35 | 0.28 | -0.32 - 0.882 | 0.01 |
|  | 40 | 0.11 | -0.51 - 0.735 | 0.04 |
|  | 45 | 0.34 | -0.27 - 0.964 | 0.00 |
|  | 50 | 1.06 | 0.377 - 1.734 | 0.00 |
|  | 55 | 0.46 | 0.00 - 0.935 | 0.37 |
|  | 60 | 0.46 | -0.06 - 0.984 | 0.02 |
|  | 65 | 0.86 | 0.197 - 1.520 | 0.82 |
|  | 70 | 1.44 | 0.508 - 2.370 | 0.02 |
|  | 75 | 2.36 | 1.136 - 3.583 | 0.16 |
|  | 80 | 4.08 | 1.852 - 6.303 | 0.00 |
| 3. Rapid Response to and Mitigation of the Spread of an Epidemic | 30 | -0.17 | -0.97 - 0.621 | 0.04 |
|  | 35 | 0.07 | -0.51 - 0.651 | 0.34 |
|  | 40 | -0.65 | -1.18 - -0.11 | 0.69 |
|  | 45 | 0.29 | -0.28 - 0.862 | 0.49 |
|  | 50 | 0.24 | -0.3.0 - 0.78 | 0.09 |
|  | 55 | 0.13 | -0.51 - 0.777 | 0.01 |
|  | 60 | 0.37 | -0.37 - 1.118 | 0.00 |
|  | 65 | 1.27 | 0.840 - 1.703 | 0.37 |
|  | 70 | 1.57 | 0.924 - 2.213 | 0.15 |
|  | 75 | 1.00 | 0.146 - 1.860 | 0.00 |
|  | 80 | -0.67 | -1.61 - 0.276 | 0.00 |
| 4. Sufficient and Robust Health System to Treat the Sick and Protect Health Workers | 20 | 0.32 | -0.17 - 0.819 | 0.01 |
|  | 25 | 0.55 | -0.05 - 1.156 | 0.06 |
|  | 30 | 0.17 | -0.50 - 0.838 | 0.36 |
|  | 35 | -0.23 | -0.76 - 0.288 | 0.01 |
|  | 40 | -0.35 | -0.87 - 0.168 | 0.08 |
|  | 45 | -0.40 | -0.88 - 0.081 | 0.01 |
|  | 50 | 0.95 | 0.292 - 1.613 | 0.00 |
|  | 55 | 0.62 | 0.090 - 1.140 | 0.15 |
|  | 60 | 0.78 | 0.198 - 1.364 | 0.01 |
|  | 65 | 0.09 | -0.57 - 0.764 | 0.00 |
|  | 70 | 3.14 | 0.525 - 5.760 | 0.05 |
| 5. Commitments to Improving National Capacity, Financing Plans to Address Gaps, and Adhering to Global Norms | 35 | -0.96 | -1.91 - -0.01 | 0.00 |
|  | 40 | -0.70 | -1.23 - -0.18 | 0.00 |
|  | 45 | -0.05 | -0.58 - 0.475 | 0.00 |
|  | 50 | 0.26 | -0.42 - 0.95 | 0.63 |
|  | 55 | 0.03 | -0.66 - 0.719 | 0.00 |
|  | 60 | -0.35 | -1.04 - 0.332 | 0.01 |
|  | 65 | 0.81 | 0.080 - 1.535 | 0.02 |
|  | 70 | 0.98 | 0.232 - 1.737 | 0.05 |
|  | 75 | 0.97 | 0.182 - 1.762 | 0.04 |
|  | 80 | 3.14 | 0.868 - 5.417 | 0.05 |
| 6. Overall Risk Environment and Country Vulnerability to Biological Threats | 50 | 0.10 | -0.74 - 0.939 | 0.29 |
|  | 55 | 0.29 | -0.27 - 0.844 | 0.00 |
|  | 60 | -0.29 | -1.15 - 0.569 | 0.00 |
|  | 65 | 0.50 | -0.12 - 1.113 | 0.00 |
|  | 70 | 1.04 | 0.459 - 1.628 | 0.05 |
|  | 75 | 0.71 | 0.126 - 1.292 | 0.05 |
|  | 80 | 0.71 | -0.24 - 1.671 | 0.02 |
|  | 85 | 1.02 | 0.342 - 1.701 | 0.00 |
