# Supplementary material for: Countries’ progress towards Global Health Security (GHS) increased health systems resilience during the Coronavirus Disease-19 (COVID-19) pandemic: A difference-in-difference study of 191 countries
Source: PLOS Glob Public Health. 2025 Jan 7;5(1):e0004051. doi: 10.1371/journal.pgph.0004051 (PMC11706378; doi:10.1371/journal.pgph.0004051)
Supplement: S4 Table — (DOCX) [file pgph.0004051.s006.docx]

**S4 Table. Difference-in-difference model results by year for overall GHSI and GHSI categories which fulfilled the parallel pre-trend assumption at varying cutoff intervals of five (2020-2022).**

| **GHSI Category** | **Cutoff value** | **Average DiD effect size (2020-2022)** | **DiD effect size for 2020** | **DiD effect size for 2021** | **DiD effect size for 2022** | ***p-value* for parallel trend** |
| --- | --- | --- | --- | --- | --- | --- |
| Overall GHSI Score (2019) | 35 | -0.79 (-1.32 - -0.27) | -0.45 (-1.27 - 0.375) | -1.14 (-2.00 - -0.27) | -0.80 (-1.76 - 0.157) | 0.19 |
|  | 40 | -0.12 (-0.65 - 0.439) | -0.23 (-1.06 - 0.613) | -0.14 (-1.11 - 0.832) | 0.04 (-0.80 - 0.881) | 0.41 |
|  | 45 | -0.51 (-1.02 - 0.011) | -0.36 (-1.07 - 0.361) | -0.56 (-1.47 - 0.358) | -0.60 (-1.37 - 0.169) | 0.51 |
|  | 60 | 1.04 (0.546 - 1.533) | 0.74 (0.242 - 1.235) | 1.61 (0.614 - 2.625) | 0.76 (0.031 - 1.488) | 0.11 |
|  | 65 | 0.97 (0.372 - 1.565) | 0.93 (0.193 - 1.669) | 1.91 (0.503 - 3.308) | 0.07 (-0.90 - 1.047) | 0.10 |
| 1. Prevention of the Emergence or Release of Pathogens | 20 | 0.41 (-0.69 - 1.519) | -0.04 (-0.85 - 0.778) | 1.05 (-2.79 - 4.911) | 0.21 (-0.74 - 1.184) | 0.16 |
|  | 30 | 0.30 (-0.32 - 0.934) | 0.18 (-0.59 - 0.971) | 0.72 (-1.03 - 2.480) | 0.00 (-0.84 - 0.859) | 0.16 |
|  | 40 | 0.56 (-0.10 - 1.230) | 0.24 (-0.51 - 0.996) | 0.90 (-0.81 - 2.629) | 0.53 (-0.41 - 1.495) | 0.43 |
|  | 70 | 1.48 (0.128 - 2.850) | 1.09 (-0.65 - 2.849) | 2.76 (0.839 - 4.687) | 0.60 (-1.72 - 2.942) | 0.75 |
|  | 75 | 1.48 (0.080 - 2.898) | 1.09 (-0.61 - 2.809) | 2.76 (0.813 - 4.713) | 0.60 (-1.70 - 2.922) | 0.75 |
| 2. Early Detection and Reporting Epidemics of Potential International Concern | 55 | 0.46 (-0.00 - 0.931) | 0.60 (0.062 - 1.146) | 0.64 (-0.37 - 1.659) | 0.14 (-0.55 - 0.844) | 0.37 |
|  | 65 | 0.85 (0.212 - 1.505) | 0.99 (0.338 - 1.654) | 1.06 (-0.29 - 2.414) | 0.51 (-0.38 - 1.423) | 0.82 |
|  | 75 | 2.35 (1.231 - 3.488) | 1.95 (0.340 - 3.565) | 4.12 (1.621 - 6.632) | 0.99 (-0.77 - 2.770) | 0.16 |
| 3. Rapid Response to and Mitigation of the Spread of an Epidemic | 35 | 0.06 (-0.47 - 0.603) | 0.26 (-0.50 - 1.034) | -0.11 (-1.01 - 0.786) | 0.04 (-0.87 - 0.969) | 0.34 |
|  | 40 | -0.65 (-1.17 - -0.12) | -0.81 (-1.52 - -0.11) | -0.96 (-1.91 - -0.00) | -0.17 (-0.97 - 0.634) | 0.69 |
|  | 45 | 0.28 (-0.21 - 0.796) | -0.29 (-1.04 - 0.455) | 0.62 (-0.44 - 1.706) | 0.53 (-0.20 - 1.277) | 0.49 |
|  | 65 | 1.27 (0.832 - 1.712) | 0.92 (0.364 - 1.476) | 1.82 (0.775 - 2.866) | 1.07 (0.309 - 1.840) | 0.37 |
|  | 70 | 1.56 (0.943 - 2.194) | 1.11 (0.273 - 1.946) | 2.36 (1.086 - 3.650) | 1.22 (0.328 - 2.130) | 0.15 |
| 4. Sufficient and Robust Health System to Treat the Sick and Protect Health Workers | 30 | 0.16 (-0.44 - 0.776) | 0.21 (-0.75 - 1.184) | 0.27 (-0.78 - 1.331) | 0.01 (-0.91 - 0.942) | 0.36 |
|  | 55 | 0.41 (-0.09 - 0.930) | -0.09 (-0.80 - 0.630) | 0.97 (-0.02 - 1.977) | 0.36 (-0.45 - 1.186) | 0.11 |
| 5. Commitments to Improving National Capacity, Financing Plans to Address Gaps, and Adhering to Global Norms | 50 | 0.26 (-0.44 - 0.971) | 0.63 (-0.03 - 1.304) | 0.29 (-1.95 - 2.540) | -0.1 (-0.97 - 0.694) | 0.63 |
| 6. Overall Risk Environment and Country Vulnerability to Biological Threats | 50 | -0.25 (-1.13 - 0.648) | -0.18 (-1.46 - 1.096) | -1.30 (-2.59 - -0.01) | 0.75 (-0.63 - 2.134) | 0.72 |
