# Supplementary material for: Countries’ progress towards Global Health Security (GHS) increased health systems resilience during the Coronavirus Disease-19 (COVID-19) pandemic: A difference-in-difference study of 191 countries
Source: PLOS Glob Public Health. 2025 Jan 7;5(1):e0004051. doi: 10.1371/journal.pgph.0004051 (PMC11706378; doi:10.1371/journal.pgph.0004051)
Supplement: S5 Table — (DOCX) [file pgph.0004051.s007.docx]

**S5 Table. Difference-in-difference model results for GHSI Category 1 (Prevention) by cutoff values (2020-2022).**

| **GHSI Category** | **Cutoff Value** | **Average DiD effect size (2020-2022)** | **95% Confidence Interval** | ***p-value* for parallel trend** |
| --- | --- | --- | --- | --- |
| 1.1 Antimicrobial resistance | 20 | -0.28 | -0.89 - 0.337 | 0.00 |
|  | 25 | -0.28 | -0.90 - 0.343 | 0.00 |
|  | 30 | -0.25 | -0.78 - 0.279 | 0.24 |
|  | 35 | 0.05 | -0.50 - 0.597 | 0.41 |
|  | 40 | 0.05 | -0.51 - 0.613 | 0.41 |
|  | 45 | 0.01 | -0.50 - 0.519 | 0.17 |
|  | 50 | 0.01 | -0.51 - 0.527 | 0.17 |
|  | 55 | -0.32 | -0.84 - 0.192 | 0.00 |
|  | 60 | 0.00 | -0.57 - 0.572 | 0.01 |
|  | 65 | 0.00 | -0.56 - 0.567 | 0.01 |
|  | 70 | 0.37 | -0.18 - 0.923 | 0.01 |
|  | 75 | 0.37 | -0.18 - 0.916 | 0.01 |
|  | 80 | 0.33 | -0.20 - 0.867 | 0.00 |
|  | 85 | 0.81 | 0.384 - 1.240 | 0.41 |
|  | 90 | 0.81 | 0.396 - 1.229 | 0.41 |
|  | 95 | 0.91 | 0.502 - 1.325 | 0.13 |
| 1.2 Zoonotic disease | 15 | 0.51 | -0.03 - 1.044 | 0.00 |
|  | 20 | 0.56 | 0.040 - 1.081 | 0.01 |
|  | 25 | -0.15 | -0.65 - 0.338 | 0.00 |
|  | 30 | -0.20 | -0.73 - 0.32 | 0.00 |
|  | 35 | -0.39 | -0.89 - 0.102 | 0.00 |
|  | 40 | -0.05 | -0.59 - 0.492 | 0.00 |
|  | 45 | -0.19 | -0.74 - 0.343 | 0.00 |
|  | 50 | 0.10 | -0.45 - 0.649 | 0.01 |
|  | 55 | -0.33 | -1.15 - 0.494 | 0.00 |
|  | 60 | -0.26 | -1.13 - 0.6 | 0.16 |
|  | 65 | 0.63 | -0.08 - 1.35 | 0.14 |
|  | 70 | 0.77 | -0.02 - 1.565 | 0.23 |
|  | 75 | 0.12 | -1.2 - 1.446 | 0.00 |
| 1.3 Biosecurity | 15 | 0.85 | -2.19 - 3.897 | 0.01 |
|  | 20 | 0.85 | -2.29 - 4.002 | 0.01 |
|  | 25 | 0.21 | -0.88 - 1.305 | 0.18 |
|  | 30 | 0.41 | -0.66 - 1.483 | 0.57 |
|  | 35 | 0.43 | -0.43 - 1.293 | 0.00 |
|  | 40 | 0.33 | -0.52 - 1.183 | 0.00 |
|  | 45 | 0.74 | 0.164 - 1.321 | 0.10 |
|  | 50 | 1.44 | 0.870 - 2.006 | 0.05 |
|  | 55 | 1.48 | 0.958 - 1.998 | 0.00 |
|  | 60 | 1.61 | 0.960 - 2.251 | 0.00 |
|  | 65 | 1.89 | 1.221 - 2.562 | 0.00 |
|  | 70 | 1.77 | 1.021 - 2.513 | 0.00 |
|  | 75 | 1.77 | 1.025 - 2.509 | 0.00 |
|  | 80 | 2.65 | 1.386 - 3.918 | 0.31 |
|  | 85 | 2.19 | 0.672 - 3.699 | 0.29 |
| 1.4 Biosafety | 15 | 0.17 | -0.58 - 0.931 | 0.18 |
|  | 20 | 0.17 | -0.6 - 0.945 | 0.18 |
|  | 25 | 0.17 | -0.61 - 0.958 | 0.18 |
|  | 30 | 0.42 | -0.19 - 1.031 | 0.05 |
|  | 35 | 0.42 | -0.17 - 1.014 | 0.05 |
|  | 40 | 0.42 | -0.18 - 1.025 | 0.05 |
|  | 45 | 0.42 | -0.19 - 1.029 | 0.05 |
|  | 50 | 0.42 | -0.21 - 1.047 | 0.05 |
|  | 55 | 0.23 | -0.39 - 0.852 | 0.00 |
|  | 60 | 0.23 | -0.37 - 0.833 | 0.00 |
|  | 65 | 0.23 | -0.37 - 0.834 | 0.00 |
|  | 70 | 0.23 | -0.33 - 0.789 | 0.00 |
|  | 75 | 0.23 | -0.37 - 0.828 | 0.00 |
|  | 80 | 0.17 | -0.49 - 0.841 | 0.02 |
|  | 85 | 0.17 | -0.42 - 0.772 | 0.02 |
|  | 90 | 0.17 | -0.5 - 0.85 | 0.02 |
|  | 95 | 0.17 | -0.45 - 0.806 | 0.02 |
| 1.5 Dual-use research and culture of responsible science | 15 | 0.89 | 0.241 - 1.541 | 0.35 |
|  | 20 | 0.89 | 0.298 - 1.483 | 0.35 |
|  | 25 | 0.89 | 0.209 - 1.573 | 0.35 |
|  | 30 | 0.89 | 0.247 - 1.535 | 0.35 |
|  | 35 | 1.31 | 0.419 - 2.195 | 0.01 |
|  | 40 | 1.31 | 0.437 - 2.177 | 0.01 |
|  | 45 | 1.31 | 0.475 - 2.139 | 0.01 |
|  | 50 | 1.31 | 0.476 - 2.138 | 0.01 |
|  | 55 | 2.92 | 1.897 - 3.937 | 0.00 |
|  | 60 | 2.92 | 1.875 - 3.959 | 0.00 |
|  | 65 | 2.92 | 1.890 - 3.944 | 0.00 |
