# Supplementary material for: Countries’ progress towards Global Health Security (GHS) increased health systems resilience during the Coronavirus Disease-19 (COVID-19) pandemic: A difference-in-difference study of 191 countries
Source: PLOS Glob Public Health. 2025 Jan 7;5(1):e0004051. doi: 10.1371/journal.pgph.0004051 (PMC11706378; doi:10.1371/journal.pgph.0004051)
Supplement: S6 Table — (DOCX) [file pgph.0004051.s008.docx]

**S6 Table. Difference-in-difference model results by year for GHSI Category 1 (Prevention) scores which fulfilled the parallel pre-trend assumption at cutoff intervals varying by five (2020-2022).**

| **GHSI Category** | **Cutoff value** | **Average DiD effect size (2020-2022)** | **DiD effect size for 2020** | **DiD effect size for 2021** | **DiD effect size for 2022** | ***p-value* for parallel trend** |
| --- | --- | --- | --- | --- | --- | --- |
| 1.1 Antimicrobial resistance | 30 | -0.25 (-0.80 - 0.292) | -0.27 (-1.02 - 0.479) | -0.19 (-1.17 - 0.781) | -0.29 (-1.28 - 0.702) | 0.24 |
|  | 35 | 0.04 (-0.50 - 0.599) | -0.06 (-0.81 - 0.689) | -0.04 (-1.05 - 0.972) | 0.24 (-0.64 - 1.133) | 0.41 |
|  | 40 | 0.04 (-0.48 - 0.574) | -0.06 (-0.83 - 0.713) | -0.04 (-1.11 - 1.035) | 0.24 (-0.62 - 1.107) | 0.41 |
|  | 45 | 0.00 (-0.49 - 0.503) | -0.36 (-1.08 - 0.368) | -0.28 (-1.30 - 0.736) | 0.66 (-0.39 - 1.716) | 0.17 |
|  | 50 | 0.00 (-0.53 - 0.543) | -0.36 (-1.07 - 0.351) | -0.28 (-1.21 - 0.651) | 0.66 (-0.36 - 1.693) | 0.17 |
|  | 85 | 0.81 (0.388 - 1.236) | 0.49 (0.094 - 0.905) | 1.30 (0.234 - 2.375) | 0.63 (-0.18 - 1.450) | 0.41 |
|  | 90 | 0.81 (0.378 - 1.246) | 0.49 (0.093 - 0.906) | 1.30 (0.220 - 2.390) | 0.63 (-0.10 - 1.374) | 0.41 |
|  | 95 | 0.91 (0.474 - 1.353) | 0.62 (0.258 - 0.988) | 1.36 (0.303 - 2.425) | 0.75 (-0.11 - 1.627) | 0.13 |
| 1.2 Zoonotic disease | 60 | -0.27 (-1.11 - 0.583) | -0.25 (-1.28 - 0.784) | -0.53 (-2.21 - 1.161) | -0.03 (-0.96 - 0.906) | 0.16 |
|  | 65 | 0.63 (-0.10 - 1.364) | 0.73 (-0.14 - 1.619) | 1.03 (-0.25 - 2.332) | 0.11 (-1.08 - 1.321) | 0.14 |
|  | 70 | 0.76 (0.019 - 1.520) | 0.61 (-0.28 - 1.520) | 1.21 (-0.14 - 2.586) | 0.47 (-0.75 - 1.708) | 0.23 |
| 1.3 Biosecurity | 25 | 0.21 (-0.90 - 1.331) | 0.04 (-0.77 - 0.868) | 1.02 (-2.57 - 4.634) | -0.44 (-1.45 - 0.569) | 0.18 |
|  | 30 | 0.40 (-0.60 - 1.420) | 0.33 (-0.49 - 1.164) | 1.22 (-2.22 - 4.682) | -0.34 (-1.34 - 0.659) | 0.57 |
|  | 45 | 0.74 (0.199 - 1.287) | 0.98 (0.344 - 1.623) | 0.92 (-0.24 - 2.090) | 0.32 (-0.65 - 1.301) | 0.10 |
|  | 80 | 2.65 (1.304 - 4.000) | 1.68 (-0.10 - 3.473) | 4.00 (1.932 - 6.069) | 2.27 (0.167 - 4.376) | 0.31 |
|  | 85 | 2.18 (0.700 - 3.671) | 1.02 (-0.95 - 2.996) | 3.09 (0.840 - 5.340) | 2.44 (0.372 - 4.518) | 0.29 |
| 1.4 Biosafety | 15 | 0.17 (-0.59 - 0.935) | -0.09 (-0.84 - 0.647) | 0.98 (-1.49 - 3.463) | -0.37 (-1.30 - 0.562) | 0.18 |
|  | 20 | 0.17 (-0.62 - 0.968) | -0.09 (-0.87 - 0.686) | 0.98 (-1.45 - 3.423) | -0.37 (-1.38 - 0.636) | 0.18 |
|  | 25 | 0.17 (-0.62 - 0.966) | -0.0 (-0.87 - 0.679) | 0.98 (-1.47 - 3.449) | -0.3 (-1.38 - 0.639) | 0.18 |
| 1.5 Dual-use research and culture of responsible science | 15 | 0.89 (0.223 - 1.559) | 0.46 (-0.68 - 1.616) | 1.14 (-0.07 - 2.360) | 1.07 (0.061 - 2.078) | 0.35 |
|  | 20 | 0.89 (0.207 - 1.575) | 0.46 (-0.59 - 1.524) | 1.14 (-0.07 - 2.356) | 1.07 (0.102 - 2.037) | 0.35 |
|  | 25 | 0.89 (0.217 - 1.565) | 0.46 (-0.64 - 1.573) | 1.14 (-0.09 - 2.375) | 1.07 (0.065 - 2.074) | 0.35 |
|  | 30 | 0.89 (0.194 - 1.588) | 0.46 (-0.63 - 1.563) | 1.14 (-0.11 - 2.390) | 1.07 (0.066 - 2.073) | 0.35 |
