# Supplementary material for: Countries’ progress towards Global Health Security (GHS) increased health systems resilience during the Coronavirus Disease-19 (COVID-19) pandemic: A difference-in-difference study of 191 countries
Source: PLOS Glob Public Health. 2025 Jan 7;5(1):e0004051. doi: 10.1371/journal.pgph.0004051 (PMC11706378; doi:10.1371/journal.pgph.0004051)
Supplement: S7 Table — (DOCX) [file pgph.0004051.s009.docx]

**S7 Table. Difference-in-difference model results for GHSI Category 2 (Early Detection) by cutoff values (2020-2022).**

| **GHSI Category** | **Cutoff Value** | **Average DiD effect size (2020-2022)** | **95% Confidence Interval** | ***p-value* for parallel trend** |
| --- | --- | --- | --- | --- |
| 2.1 Laboratory systems strength and quality | 30 | 0.08 | -0.43 - 0.603 | 0.00 |
|  | 35 | 0.08 | -0.45 - 0.617 | 0.00 |
|  | 40 | -0.14 | -0.74 - 0.441 | 0.06 |
|  | 45 | -0.14 | -0.71 - 0.416 | 0.06 |
|  | 50 | -0.14 | -0.7 - 0.404 | 0.06 |
|  | 55 | -0.08 | -0.64 - 0.476 | 0.00 |
|  | 60 | -0.08 | -0.59 - 0.425 | 0.00 |
|  | 65 | -0.13 | -0.74 - 0.476 | 0.03 |
|  | 70 | -0.13 | -0.75 - 0.491 | 0.03 |
|  | 75 | -0.13 | -0.75 - 0.485 | 0.03 |
|  | 80 | 0.85 | -0.09 - 1.787 | 0.00 |
|  | 85 | 0.85 | -0.11 - 1.8 | 0.00 |
| 2.2 Laboratory supply chains | 15 | -0.13 | -1.33 - 1.052 | 0.51 |
|  | 20 | -0.13 | -1.3 - 1.022 | 0.51 |
|  | 25 | -0.13 | -1.31 - 1.037 | 0.51 |
|  | 30 | -0.13 | -1.32 - 1.047 | 0.51 |
|  | 35 | -0.13 | -1.34 - 1.066 | 0.51 |
|  | 40 | -0.13 | -1.33 - 1.054 | 0.51 |
|  | 45 | -0.13 | -1.35 - 1.074 | 0.51 |
|  | 50 | -0.13 | -1.29 - 1.02 | 0.51 |
|  | 55 | 4.08 | 2.037 - 6.119 | 0.00 |
|  | 60 | 4.08 | 1.829 - 6.327 | 0.00 |
|  | 65 | 4.08 | 1.839 - 6.317 | 0.00 |
|  | 70 | 4.08 | 1.893 - 6.263 | 0.00 |
|  | 75 | 4.08 | 1.755 - 6.401 | 0.00 |
|  | 80 | 4.08 | 1.936 - 6.220 | 0.00 |
|  | 85 | 4.08 | 1.926 - 6.230 | 0.00 |
|  | 90 | 4.08 | 1.896 - 6.260 | 0.00 |
|  | 95 | 4.08 | 1.698 - 6.458 | 0.00 |
| 2.3 Real-time surveillance and reporting | 15 | 1.03 | 0.398 - 1.671 | 0.57 |
|  | 20 | 1.03 | 0.385 - 1.684 | 0.57 |
|  | 25 | 1.03 | 0.402 - 1.667 | 0.57 |
|  | 30 | 0.75 | 0.206 - 1.302 | 0.00 |
|  | 35 | 0.75 | 0.189 - 1.319 | 0.00 |
|  | 40 | 0.77 | 0.204 - 1.339 | 0.00 |
|  | 45 | 0.77 | 0.229 - 1.314 | 0.00 |
|  | 50 | 0.77 | 0.239 - 1.304 | 0.00 |
|  | 55 | 0.80 | 0.231 - 1.366 | 0.00 |
|  | 60 | 0.80 | 0.210 - 1.387 | 0.00 |
|  | 65 | 1.47 | 0.789 - 2.154 | 0.00 |
|  | 70 | 1.47 | 0.790 - 2.153 | 0.00 |
|  | 75 | 1.47 | 0.793 - 2.150 | 0.00 |
|  | 80 | 1.89 | 1.057 - 2.720 | 0.00 |
|  | 85 | 1.89 | 1.060 - 2.717 | 0.00 |
|  | 90 | 1.40 | 0.764 - 2.045 | 0.00 |
|  | 95 | 1.40 | 0.756 - 2.053 | 0.00 |
| 2.4 Surveillance data accessibility and transparency | 15 | -0.38 | -0.88 - 0.11 | 0.00 |
|  | 20 | -0.38 | -0.87 - 0.098 | 0.00 |
|  | 25 | -1.38 | -2.1 - -0.67 | 0.00 |
|  | 30 | -1.37 | -2.06 - -0.69 | 0.00 |
|  | 35 | -0.62 | -1.21 - -0.02 | 0.02 |
|  | 40 | -0.70 | -1.24 - -0.15 | 0.04 |
|  | 45 | 0.10 | -0.65 - 0.841 | 0.10 |
|  | 50 | 0.23 | -0.53 - 0.983 | 0.03 |
|  | 55 | -0.44 | -1.01 - 0.121 | 0.16 |
|  | 60 | -0.61 | -1.19 - -0.03 | 0.02 |
|  | 65 | -0.48 | -1.07 - 0.1 | 0.02 |
|  | 70 | -0.48 | -1.04 - 0.07 | 0.02 |
|  | 75 | 0.08 | -0.46 - 0.618 | 0.01 |
|  | 80 | -0.04 | -0.59 - 0.505 | 0.33 |
|  | 85 | -0.34 | -0.99 - 0.301 | 0.52 |
|  | 90 | -0.61 | -1.27 - 0.031 | 0.66 |
|  | 95 | -0.93 | -1.76 - -0.1 | 0.01 |
| 2.5 Case-based investigation | 15 | -0.38 | -1.01 - 0.243 | 0.03 |
|  | 20 | -0.38 | -1.1 - 0.334 | 0.03 |
|  | 25 | -0.38 | -1.11 - 0.336 | 0.03 |
|  | 30 | 0.41 | -0.53 - 1.36 | 0.01 |
|  | 35 | 0.41 | -0.48 - 1.306 | 0.01 |
|  | 40 | 0.89 | -0.22 - 2 | 0.02 |
|  | 45 | 0.89 | -0.18 - 1.968 | 0.02 |
|  | 50 | 0.89 | -0.21 - 1.992 | 0.02 |
|  | 55 | 0.15 | -0.73 - 1.028 | 0.15 |
|  | 60 | 0.15 | -0.68 - 0.98 | 0.15 |
|  | 65 | 0.80 | -0.34 - 1.946 | 0.15 |
|  | 70 | 0.80 | -0.29 - 1.897 | 0.15 |
|  | 75 | 0.80 | -0.31 - 1.921 | 0.15 |
|  | 80 | -0.24 | -2.1 - 1.614 | 0.01 |
|  | 85 | -0.24 | -2.1 - 1.614 | 0.01 |
|  | 90 | -0.24 | -2.09 - 1.605 | 0.01 |
|  | 95 | -0.24 | -2.09 - 1.604 | 0.01 |
| 2.6 Epidemiology workforce | 15 | -1.28 | -1.9 - -0.66 | 0.00 |
|  | 20 | -1.28 | -1.93 - -0.63 | 0.00 |
|  | 25 | -1.28 | -1.95 - -0.61 | 0.00 |
|  | 30 | -0.43 | -0.95 - 0.09 | 0.42 |
|  | 35 | -0.43 | -0.93 - 0.068 | 0.42 |
|  | 40 | -0.43 | -0.94 - 0.081 | 0.42 |
|  | 45 | -0.43 | -0.93 - 0.07 | 0.42 |
|  | 50 | -0.43 | -0.97 - 0.107 | 0.42 |
|  | 55 | -0.48 | -1.13 - 0.165 | 0.05 |
|  | 60 | -0.48 | -1.17 - 0.203 | 0.05 |
|  | 65 | -0.48 | -1.1 - 0.138 | 0.05 |
|  | 70 | -0.48 | -1.14 - 0.171 | 0.05 |
|  | 75 | -0.48 | -1.1 - 0.135 | 0.05 |
|  | 80 | -0.41 | -1.1 - 0.276 | 0.34 |
|  | 85 | -0.41 | -1.13 - 0.305 | 0.34 |
|  | 90 | -0.41 | -1.05 - 0.224 | 0.34 |
|  | 95 | -0.41 | -1.16 - 0.335 | 0.34 |
