# Supplementary material for: Countries’ progress towards Global Health Security (GHS) increased health systems resilience during the Coronavirus Disease-19 (COVID-19) pandemic: A difference-in-difference study of 191 countries
Source: PLOS Glob Public Health. 2025 Jan 7;5(1):e0004051. doi: 10.1371/journal.pgph.0004051 (PMC11706378; doi:10.1371/journal.pgph.0004051)
Supplement: S8 Table — (DOCX) [file pgph.0004051.s010.docx]

**S8 Table. Difference-in-difference model results by year for GHSI Category 2 (Early Detection) scores which fulfilled the parallel pre-trend assumption at cutoff intervals varying by five (2020-2022).**

| **GHSI Category** | **Cutoff value** | **Average DiD effect size (2020-2022)** | **DiD effect size for 2020** | **DiD effect size for 2021** | **DiD effect size for 2022** | ***p-value* for parallel trend** |
| --- | --- | --- | --- | --- | --- | --- |
| 2.2 Laboratory supply chains | 15 | -0.14 (-1.25 - 0.97) | -0.12 (-1.7 - 1.45) | -0.03 (-1.67 - 1.61) | -0.27 (-1.91 - 1.37) | 0.51 |
|  | 20 | -0.14 (-1.31 - 1.03) | -0.12 (-1.7 - 1.45) | -0.03 (-1.53 - 1.47) | -0.27 (-1.83 - 1.29) | 0.51 |
|  | 25 | -0.14 (-1.32 - 1.04) | -0.12 (-1.68 - 1.43) | -0.03 (-1.61 - 1.56) | -0.27 (-1.9 - 1.37) | 0.51 |
|  | 30 | -0.14 (-1.32 - 1.04) | -0.12 (-1.66 - 1.41) | -0.03 (-1.55 - 1.5) | -0.27 (-1.85 - 1.32) | 0.51 |
|  | 35 | -0.14 (-1.35 - 1.08) | -0.12 (-1.68 - 1.43) | -0.03 (-1.6 - 1.55) | -0.27 (-1.89 - 1.36) | 0.51 |
|  | 40 | -0.14 (-1.32 - 1.04) | -0.12 (-1.69 - 1.45) | -0.03 (-1.5 - 1.44) | -0.27 (-1.87 - 1.34) | 0.51 |
|  | 45 | -0.14 (-1.36 - 1.08) | -0.12 (-1.72 - 1.47) | -0.03 (-1.59 - 1.54) | -0.27 (-1.93 - 1.39) | 0.51 |
|  | 50 | -0.14 (-1.31 - 1.03) | -0.12 (-1.72 - 1.47) | -0.03 (-1.58 - 1.53) | -0.27 (-1.9 - 1.37) | 0.51 |
| 2.3 Real-time surveillance and reporting | 15 | 1.03 (0.4 - 1.67) | -0.09 (-0.81 - 0.62) | 2.36 (0.66 - 4.06) | 0.84 (-0.07 - 1.74) | 0.57 |
|  | 20 | 1.03 (0.32 - 1.75) | -0.09 (-0.81 - 0.63) | 2.36 (0.71 - 4.01) | 0.84 (-0.08 - 1.76) | 0.57 |
|  | 25 | 1.03 (0.37 - 1.7) | -0.09 (-0.83 - 0.65) | 2.36 (0.74 - 3.98) | 0.84 (-0.02 - 1.7) | 0.57 |
| 2.4 Surveillance data accessibility and transparency | 45 | 0.1 (-0.64 - 0.83) | -0.31 (-1.04 - 0.41) | 0.69 (-1.66 - 3.05) | -0.09 (-0.95 - 0.77) | 0.10 |
|  | 55 | -0.45 (-1.06 - 0.16) | -0.65 (-1.36 - 0.07) | 0.19 (-1.17 - 1.55) | -0.88 (-1.67 - -0.1) | 0.16 |
|  | 80 | -0.05 (-0.57 - 0.48) | -0.42 (-1.13 - 0.3) | 0.67 (-0.49 - 1.83) | -0.40 (-1.25 - 0.46) | 0.33 |
|  | 85 | -0.35 (-0.98 - 0.28) | -0.71 (-1.65 - 0.22) | 0.48 (-0.67 - 1.62) | -0.81 (-1.86 - 0.24) | 0.52 |
|  | 90 | -0.62 (-1.3 - 0.06) | -0.99 (-1.94 - -0.05) | 0.12 (-1.06 - 1.3) | -0.99 (-2.17 - 0.2) | 0.66 |
| 2.5 Case-based investigation | 60 | 0.15 (-0.71 - 1) | 0.68 (-0.48 - 1.83) | -0.28 (-1.75 - 1.2) | 0.04 (-1.29 - 1.38) | 0.15 |
|  | 65 | 0.80 (-0.32 - 1.92) | 1.06 (-0.59 - 2.71) | 0.55 (-0.85 - 1.95) | 0.80 (-0.8 - 2.4) | 0.15 |
|  | 70 | 0.80 (-0.33 - 1.94) | 1.06 (-0.7 - 2.82) | 0.55 (-1.02 - 2.12) | 0.80 (-0.79 - 2.38) | 0.15 |
|  | 75 | 0.80 (-0.23 - 1.83) | 1.06 (-0.59 - 2.71) | 0.55 (-0.96 - 2.07) | 0.80 (-0.78 - 2.37) | 0.15 |
| 2.6 Epidemiology workforce | 30 | -0.43 (-0.97 - 0.1) | 0.30 (-0.37 - 0.98) | -0.97 (-1.86 - -0.09) | -0.63 (-1.64 - 0.39) | 0.42 |
|  | 35 | -0.43 (-0.97 - 0.11) | 0.30 (-0.34 - 0.95) | -0.97 (-1.84 - -0.11) | -0.63 (-1.55 - 0.3) | 0.42 |
|  | 40 | -0.43 (-0.95 - 0.08) | 0.30 (-0.37 - 0.97) | -0.97 (-1.91 - -0.04) | -0.63 (-1.62 - 0.36) | 0.42 |
|  | 45 | -0.43 (-0.94 - 0.07) | 0.30 (-0.36 - 0.97) | -0.97 (-1.93 - -0.02) | -0.63 (-1.61 - 0.36) | 0.42 |
|  | 50 | -0.43 (-0.98 - 0.12) | 0.30 (-0.4 - 1) | -0.97 (-1.9 - -0.05) | -0.63 (-1.64 - 0.39) | 0.42 |
|  | 80 | -0.42 (-1.12 - 0.28) | 1.11 (0.39 - 1.84) | -1.29 (-2.83 - 0.24) | -1.07 (-2.24 - 0.11) | 0.34 |
|  | 85 | -0.42 (-1.11 - 0.28) | 1.11 (0.4 - 1.82) | -1.29 (-2.83 - 0.24) | -1.07 (-2.3 - 0.16) | 0.34 |
|  | 90 | -0.42 (-1.09 - 0.26) | 1.11 (0.39 - 1.84) | -1.29 (-2.73 - 0.14) | -1.07 (-2.34 - 0.21) | 0.34 |
|  | 95 | -0.42 (-1.12 - 0.29) | 1.11 (0.35 - 1.88) | -1.29 (-2.84 - 0.25) | -1.07 (-2.28 - 0.15) | 0.34 |
