# Supplementary material for: Countries’ progress towards Global Health Security (GHS) increased health systems resilience during the Coronavirus Disease-19 (COVID-19) pandemic: A difference-in-difference study of 191 countries
Source: PLOS Glob Public Health. 2025 Jan 7;5(1):e0004051. doi: 10.1371/journal.pgph.0004051 (PMC11706378; doi:10.1371/journal.pgph.0004051)
Supplement: S9 Table — (DOCX) [file pgph.0004051.s011.docx]

**S9 Table. Difference-in-difference model results for GHSI Category 3 (Rapid Response) by cutoff values (2020-2022).**

| **GHSI Category** | **Cutoff Value** | **Average DiD effect size (2020-2022)** | **95% Confidence Interval** | ***p-value* for parallel trend** |
| --- | --- | --- | --- | --- |
| 3.1 Emergency preparedness and response planning | 15 | -0.51 | -1.00 - -0.02 | 0.18 |
|  | 20 | -0.61 | -1.17 - -0.05 | 0.34 |
|  | 25 | -0.32 | -0.83 - 0.184 | 0.43 |
|  | 30 | -0.28 | -0.82 - 0.259 | 0.45 |
|  | 35 | -0.54 | -1.09 - 0.008 | 0.03 |
|  | 40 | -0.83 | -1.37 - -0.28 | 0.02 |
|  | 45 | -0.99 | -1.56 - -0.43 | 0.32 |
|  | 50 | -0.99 | -1.56 - -0.42 | 0.32 |
|  | 55 | -0.01 | -0.61 - 0.583 | 0.00 |
|  | 60 | 0.84 | 0.107 - 1.567 | 0.02 |
|  | 65 | 1.32 | 0.568 - 2.073 | 0.00 |
|  | 70 | 0.87 | 0.074 - 1.668 | 0.01 |
|  | 75 | 0.87 | 0.125 - 1.616 | 0.01 |
|  | 80 | 0.87 | 0.075 - 1.666 | 0.01 |
|  | 85 | 1.82 | 0.651 - 2.995 | 0.06 |
|  | 90 | 1.82 | 0.648 - 2.999 | 0.06 |
|  | 95 | 3.05 | 1.744 - 4.359 | 0.02 |
| 3.2 Exercising response plans | 15 | 1.17 | 0.586 - 1.750 | 0.02 |
|  | 20 | 1.17 | 0.567 - 1.769 | 0.02 |
|  | 25 | 1.17 | 0.558 - 1.778 | 0.02 |
|  | 30 | -0.59 | -1.32 - 0.143 | 0.03 |
|  | 35 | -0.59 | -1.36 - 0.183 | 0.03 |
|  | 40 | -1.26 | -2.78 - 0.264 | 0.13 |
|  | 45 | -1.26 | -2.81 - 0.287 | 0.13 |
|  | 50 | -1.26 | -2.86 - 0.342 | 0.13 |
|  | 55 | -1.26 | -2.74 - 0.218 | 0.13 |
|  | 60 | -1.26 | -2.79 - 0.267 | 0.13 |
|  | 65 | -1.26 | -2.81 - 0.294 | 0.13 |
|  | 70 | -1.26 | -2.87 - 0.352 | 0.13 |
|  | 75 | -1.26 | -2.76 - 0.244 | 0.13 |
|  | 80 | 1.57 | 0.474 - 2.661 | 0.01 |
|  | 85 | 1.57 | 0.447 - 2.688 | 0.01 |
| 3.3 Emergency response operation | 15 | 0.35 | -0.23 - 0.928 | 0.00 |
|  | 20 | 0.35 | -0.29 - 0.989 | 0.00 |
|  | 25 | 0.35 | -0.28 - 0.979 | 0.00 |
|  | 30 | 0.35 | -0.23 - 0.93 | 0.00 |
|  | 35 | 1.34 | 0.615 - 2.061 | 0.00 |
|  | 40 | 1.34 | 0.583 - 2.093 | 0.00 |
|  | 45 | 1.34 | 0.612 - 2.064 | 0.00 |
|  | 50 | 1.34 | 0.623 - 2.053 | 0.00 |
|  | 55 | 1.34 | 0.607 - 2.069 | 0.00 |
|  | 60 | 1.34 | 0.621 - 2.055 | 0.00 |
|  | 65 | 1.34 | 0.647 - 2.029 | 0.00 |
|  | 70 | 0.08 | -0.43 - 0.589 | 0.00 |
|  | 75 | 0.08 | -0.42 - 0.578 | 0.00 |
|  | 80 | 0.08 | -0.47 - 0.629 | 0.00 |
|  | 85 | 0.08 | -0.43 - 0.59 | 0.00 |
|  | 90 | 0.08 | -0.41 - 0.57 | 0.00 |
|  | 95 | 0.08 | -0.44 - 0.593 | 0.00 |
| 3.4 Linking public health and security authorities | 15 | 0.24 | -0.34 - 0.834 | 0.52 |
|  | 20 | 0.24 | -0.33 - 0.822 | 0.52 |
|  | 25 | 0.24 | -0.32 - 0.81 | 0.52 |
|  | 30 | 0.24 | -0.36 - 0.851 | 0.52 |
|  | 35 | 0.24 | -0.33 - 0.819 | 0.52 |
|  | 40 | 0.24 | -0.36 - 0.85 | 0.52 |
|  | 45 | 0.24 | -0.32 - 0.816 | 0.52 |
|  | 50 | 0.24 | -0.32 - 0.815 | 0.52 |
|  | 55 | 0.24 | -0.33 - 0.826 | 0.52 |
|  | 60 | 0.24 | -0.34 - 0.834 | 0.52 |
|  | 65 | 0.24 | -0.31 - 0.803 | 0.52 |
|  | 70 | 0.24 | -0.33 - 0.819 | 0.52 |
|  | 75 | 0.24 | -0.31 - 0.801 | 0.52 |
|  | 80 | 0.24 | -0.34 - 0.831 | 0.52 |
|  | 85 | 0.24 | -0.34 - 0.83 | 0.52 |
|  | 90 | 0.24 | -0.32 - 0.813 | 0.52 |
|  | 95 | 0.24 | -0.36 - 0.848 | 0.52 |
| 3.5 Risk communication | 40 | -0.37 | -0.95 - 0.214 | 0.04 |
|  | 45 | -0.05 | -0.61 - 0.513 | 0.02 |
|  | 50 | -0.05 | -0.58 - 0.488 | 0.02 |
|  | 55 | 0.14 | -0.36 - 0.646 | 0.01 |
|  | 60 | 0.29 | -0.2 - 0.782 | 0.00 |
|  | 65 | 0.29 | -0.21 - 0.799 | 0.00 |
|  | 70 | 0.54 | -0.03 - 1.122 | 0.00 |
|  | 75 | 0.60 | -0.02 - 1.223 | 0.00 |
|  | 80 | 0.86 | 0.244 - 1.473 | 0.00 |
|  | 85 | 0.89 | -0.52 - 2.316 | 0.03 |
|  | 90 | 1.15 | -0.63 - 2.931 | 0.01 |
|  | 95 | 1.15 | -0.38 - 2.676 | 0.01 |
| 3.6 Access to communications infrastructure | 65 | 3.02 | 1.026 - 5.010 | 0.50 |
|  | 70 | 0.96 | 0.373 - 1.554 | 0.09 |
|  | 75 | 0.73 | 0.204 - 1.260 | 0.03 |
|  | 80 | 1.00 | 0.472 - 1.528 | 0.00 |
|  | 85 | 0.00 | -0.75 - 0.747 | 0.05 |
|  | 90 | -1.59 | -5.24 - 2.05 | 0.09 |
|  | 95 | -1.55 | -4.23 - 1.118 | 0.19 |
| 3.7 Trade and travel restrictions | 80 | 0.84 | 0.002 - 1.685 | 0.02 |
|  | 85 | 0.84 | 0.055 - 1.632 | 0.02 |
|  | 90 | 0.84 | 0.071 - 1.616 | 0.02 |
|  | 95 | 0.84 | 0.007 - 1.680 | 0.02 |
