# Supplementary material for: Countries’ progress towards Global Health Security (GHS) increased health systems resilience during the Coronavirus Disease-19 (COVID-19) pandemic: A difference-in-difference study of 191 countries
Source: PLOS Glob Public Health. 2025 Jan 7;5(1):e0004051. doi: 10.1371/journal.pgph.0004051 (PMC11706378; doi:10.1371/journal.pgph.0004051)
Supplement: S10 Table — (DOCX) [file pgph.0004051.s012.docx]

**S10 Table. Difference-in-difference model results by year for GHSI Category 3 (Rapid Response) scores which fulfilled the parallel pre-trend assumption at cutoff intervals varying by five (2020-2022).**

| **GHSI Category** | **Cutoff value** | **Average DiD effect size (2020-2022)** | **DiD effect size for 2020** | **DiD effect size for 2021** | **DiD effect size for 2022** | ***p-value* for parallel trend** |
| --- | --- | --- | --- | --- | --- | --- |
| 3.1 Emergency preparedness and response planning | 15 | -0.51 (-1.05 - 0.02) | -1.02 (-1.74 - -0.3) | -0.8 (-1.81 - 0.22) | 0.27 (-0.58 - 1.13) | 0.18 |
|  | 20 | -0.61 (-1.15 - -0.08) | -1.13 (-1.81 - -0.46) | -0.67 (-1.67 - 0.32) | -0.03 (-0.78 - 0.72) | 0.34 |
|  | 25 | -0.33 (-0.87 - 0.21) | -0.61 (-1.33 - 0.11) | -0.46 (-1.44 - 0.51) | 0.09 (-0.79 - 0.97) | 0.43 |
|  | 30 | -0.28 (-0.81 - 0.24) | -0.74 (-1.55 - 0.06) | -0.42 (-1.55 - 0.7) | 0.32 (-0.62 - 1.26) | 0.45 |
|  | 45 | -1.00 (-1.58 - -0.42) | -0.78 (-1.54 - -0.01) | -1.62 (-2.81 - -0.43) | -0.60 (-1.4 - 0.19) | 0.32 |
|  | 50 | -1.00 (-1.56 - -0.43) | -0.78 (-1.48 - -0.07) | -1.62 (-2.95 - -0.29) | -0.60 (-1.38 - 0.17) | 0.32 |
| 3.2 Exercising response plans | 40 | -1.26 (-2.8 - 0.28) | -2.34 (-5.15 - 0.48) | -1.48 (-4.49 - 1.52) | 0.03 (-2.14 - 2.21) | 0.13 |
|  | 45 | -1.26 (-2.79 - 0.27) | -2.34 (-5.1 - 0.43) | -1.48 (-4.55 - 1.58) | 0.03 (-2.03 - 2.1) | 0.13 |
|  | 50 | -1.26 (-2.81 - 0.28) | -2.34 (-4.93 - 0.25) | -1.48 (-4.52 - 1.55) | 0.03 (-2.08 - 2.15) | 0.13 |
|  | 55 | -1.26 (-2.79 - 0.26) | -2.34 (-4.89 - 0.22) | -1.48 (-4.37 - 1.4) | 0.03 (-2.09 - 2.16) | 0.13 |
|  | 60 | -1.26 (-2.8 - 0.27) | -2.34 (-5.03 - 0.36) | -1.48 (-4.33 - 1.36) | 0.03 (-2.06 - 2.13) | 0.13 |
|  | 65 | -1.26 (-2.82 - 0.3) | -2.34 (-5.09 - 0.41) | -1.48 (-4.45 - 1.48) | 0.03 (-2.01 - 2.07) | 0.13 |
|  | 70 | -1.26 (-2.84 - 0.32) | -2.34 (-5.1 - 0.42) | -1.48 (-4.55 - 1.58) | 0.03 (-2.17 - 2.24) | 0.13 |
|  | 75 | -1.26 (-2.9 - 0.37) | -2.34 (-4.85 - 0.18) | -1.48 (-4.43 - 1.46) | 0.03 (-2.01 - 2.07) | 0.13 |
| 3.4 Linking public health and security authorities | 15 | 0.24 (-0.32 - 0.81) | 0.09 (-0.63 - 0.82) | 0.26 (-0.84 - 1.36) | 0.38 (-0.44 - 1.21) | 0.52 |
|  | 20 | 0.24 (-0.29 - 0.78) | 0.09 (-0.72 - 0.91) | 0.26 (-1.01 - 1.52) | 0.38 (-0.49 - 1.25) | 0.52 |
|  | 25 | 0.24 (-0.32 - 0.81) | 0.09 (-0.71 - 0.89) | 0.26 (-0.96 - 1.48) | 0.38 (-0.5 - 1.26) | 0.52 |
|  | 30 | 0.24 (-0.32 - 0.81) | 0.09 (-0.73 - 0.91) | 0.26 (-0.94 - 1.46) | 0.38 (-0.44 - 1.2) | 0.52 |
|  | 35 | 0.24 (-0.31 - 0.8) | 0.09 (-0.73 - 0.91) | 0.26 (-0.96 - 1.47) | 0.38 (-0.5 - 1.27) | 0.52 |
|  | 40 | 0.24 (-0.35 - 0.84) | 0.09 (-0.68 - 0.86) | 0.26 (-0.93 - 1.45) | 0.38 (-0.49 - 1.25) | 0.52 |
|  | 45 | 0.24 (-0.35 - 0.84) | 0.09 (-0.68 - 0.87) | 0.26 (-0.98 - 1.49) | 0.38 (-0.54 - 1.3) | 0.52 |
|  | 50 | 0.24 (-0.29 - 0.78) | 0.09 (-0.69 - 0.88) | 0.26 (-0.92 - 1.43) | 0.38 (-0.46 - 1.22) | 0.52 |
|  | 55 | 0.24 (-0.29 - 0.78) | 0.09 (-0.66 - 0.85) | 0.26 (-0.89 - 1.4) | 0.38 (-0.54 - 1.31) | 0.52 |
|  | 60 | 0.24 (-0.34 - 0.83) | 0.09 (-0.65 - 0.83) | 0.26 (-0.96 - 1.47) | 0.38 (-0.43 - 1.19) | 0.52 |
|  | 65 | 0.24 (-0.34 - 0.82) | 0.09 (-0.65 - 0.83) | 0.26 (-0.9 - 1.41) | 0.38 (-0.47 - 1.23) | 0.52 |
|  | 70 | 0.24 (-0.34 - 0.83) | 0.09 (-0.71 - 0.9) | 0.26 (-0.95 - 1.47) | 0.38 (-0.48 - 1.24) | 0.52 |
|  | 75 | 0.24 (-0.34 - 0.83) | 0.09 (-0.67 - 0.85) | 0.26 (-0.94 - 1.45) | 0.38 (-0.53 - 1.29) | 0.52 |
|  | 80 | 0.24 (-0.34 - 0.82) | 0.09 (-0.7 - 0.89) | 0.26 (-0.91 - 1.43) | 0.38 (-0.45 - 1.22) | 0.52 |
|  | 85 | 0.24 (-0.32 - 0.81) | 0.09 (-0.66 - 0.85) | 0.26 (-0.92 - 1.44) | 0.38 (-0.51 - 1.27) | 0.52 |
|  | 90 | 0.24 (-0.35 - 0.84) | 0.09 (-0.64 - 0.82) | 0.26 (-0.89 - 1.41) | 0.38 (-0.39 - 1.16) | 0.52 |
|  | 95 | 0.24 (-0.29 - 0.78) | 0.09 (-0.66 - 0.84) | 0.26 (-0.99 - 1.5) | 0.38 (-0.45 - 1.22) | 0.52 |
