# Supplementary material for: Countries’ progress towards Global Health Security (GHS) increased health systems resilience during the Coronavirus Disease-19 (COVID-19) pandemic: A difference-in-difference study of 191 countries
Source: PLOS Glob Public Health. 2025 Jan 7;5(1):e0004051. doi: 10.1371/journal.pgph.0004051 (PMC11706378; doi:10.1371/journal.pgph.0004051)
Supplement: S11 Table — (DOCX) [file pgph.0004051.s013.docx]

**S11 Table. Difference-in-difference model results for GHSI Category 4 (Health System) by cutoff values (2020-2022).**

| **GHSI Category** | **Cutoff Value** | **Average DiD effect size (2020-2022)** | **95% Confidence Interval** | ***p-value* for parallel trend** |
| --- | --- | --- | --- | --- |
| 4.1 Health capacity in clinics, hospitals, and community care centers | 15 | -0.32 | -0.89 - 0.235 | 0.00 |
|  | 20 | 0.01 | -0.55 - 0.577 | 0.03 |
|  | 25 | -0.01 | -0.62 - 0.584 | 0.25 |
|  | 30 | 0.43 | -0.11 - 0.979 | 0.28 |
|  | 35 | 0.66 | 0.155 - 1.174 | 0.02 |
|  | 40 | 0.48 | -0.18 - 1.14 | 0.01 |
|  | 45 | 0.42 | -0.1 - 0.948 | 0.04 |
|  | 50 | 0.17 | -0.5 - 0.849 | 0.09 |
|  | 55 | 1.14 | 0.605 - 1.677 | 0.22 |
|  | 60 | 1.42 | 0.490 - 2.356 | 0.01 |
|  | 65 | 1.61 | 0.275 - 2.943 | 0.03 |
|  | 70 | 1.61 | 0.247 - 2.971 | 0.03 |
| 4.2 Supply chain for health system and healthcare workers | 15 | 0.10 | -0.61 - 0.812 | 0.00 |
|  | 20 | -0.11 | -0.69 - 0.46 | 0.00 |
|  | 25 | 0.05 | -0.45 - 0.547 | 0.00 |
|  | 30 | -0.08 | -0.68 - 0.504 | 0.00 |
|  | 35 | 0.45 | -0.04 - 0.936 | 0.01 |
|  | 40 | 0.46 | -0.15 - 1.079 | 0.00 |
|  | 45 | 0.07 | -0.49 - 0.628 | 0.00 |
|  | 50 | 0.07 | -0.49 - 0.626 | 0.00 |
|  | 55 | -0.29 | -0.86 - 0.272 | 0.00 |
|  | 60 | -0.10 | -0.62 - 0.412 | 0.04 |
|  | 65 | 0.07 | -0.6 - 0.754 | 0.00 |
|  | 70 | 0.27 | -0.54 - 1.075 | 0.01 |
|  | 75 | 0.86 | 0.107 - 1.612 | 0.33 |
|  | 80 | 0.86 | 0.115 - 1.603 | 0.33 |
| 4.3 Medical countermeasures and personnel deployment | 15 | -0.63 | -1.19 - -0.07 | 0.40 |
|  | 20 | -0.63 | -1.18 - -0.08 | 0.40 |
|  | 25 | -0.63 | -1.16 - -0.1 | 0.40 |
|  | 30 | -0.63 | -1.15 - -0.11 | 0.40 |
|  | 35 | -0.63 | -1.15 - -0.12 | 0.40 |
|  | 40 | -0.63 | -1.17 - -0.1 | 0.40 |
|  | 45 | -0.63 | -1.18 - -0.08 | 0.40 |
|  | 50 | -0.63 | -1.19 - -0.07 | 0.40 |
|  | 55 | 2.14 | 1.223 - 3.050 | 0.36 |
|  | 60 | 2.14 | 1.243 - 3.029 | 0.36 |
|  | 65 | 2.14 | 1.269 - 3.003 | 0.36 |
|  | 70 | 2.14 | 1.189 - 3.083 | 0.36 |
|  | 75 | 2.14 | 1.208 - 3.065 | 0.36 |
|  | 80 | 2.14 | 1.214 - 3.058 | 0.36 |
|  | 85 | 2.14 | 1.248 - 3.024 | 0.36 |
|  | 90 | 2.14 | 1.286 - 2.986 | 0.36 |
|  | 95 | 2.14 | 1.253 - 3.019 | 0.36 |
| 4.4 Healthcare access | 50 | -2.82 | -3.55 - -2.09 | 0.00 |
|  | 55 | 0.64 | -0.05 - 1.337 | 0.01 |
|  | 60 | -0.21 | -0.84 - 0.422 | 0.13 |
|  | 65 | 1.91 | 1.072 - 2.740 | 0.00 |
|  | 70 | 0.69 | -0.47 - 1.856 | 0.09 |
|  | 75 | 0.69 | -0.59 - 1.982 | 0.09 |
|  | 80 | 0.69 | -0.42 - 1.81 | 0.09 |
|  | 85 | 0.69 | -0.49 - 1.883 | 0.09 |
|  | 90 | 0.97 | -0.58 - 2.521 | 0.05 |
|  | 95 | 1.07 | -1.24 - 3.389 | 0.03 |
| 4.5 Communications with healthcare workers during a public health emergency | 15 | 0.28 | -0.41 - 0.985 | 0.20 |
|  | 20 | 0.28 | -0.39 - 0.964 | 0.20 |
|  | 25 | 0.28 | -0.4 - 0.973 | 0.20 |
|  | 30 | 0.28 | -0.36 - 0.93 | 0.20 |
|  | 35 | 0.28 | -0.38 - 0.953 | 0.20 |
|  | 40 | 0.28 | -0.37 - 0.936 | 0.20 |
|  | 45 | 0.28 | -0.36 - 0.933 | 0.20 |
|  | 50 | 0.28 | -0.37 - 0.937 | 0.20 |
|  | 55 | 0.48 | -0.16 - 1.126 | 0.06 |
|  | 60 | 0.48 | -0.13 - 1.096 | 0.06 |
|  | 65 | 0.48 | -0.15 - 1.118 | 0.06 |
|  | 70 | 0.48 | -0.13 - 1.095 | 0.06 |
|  | 75 | 0.48 | -0.13 - 1.094 | 0.06 |
|  | 80 | 0.48 | -0.11 - 1.077 | 0.06 |
|  | 85 | 0.48 | -0.1 - 1.067 | 0.06 |
|  | 90 | 0.48 | -0.14 - 1.109 | 0.06 |
|  | 95 | 0.48 | -0.18 - 1.14 | 0.06 |
| 4.6 Infection control practices | 15 | 0.53 | 0 - 1.066 | 0.04 |
|  | 20 | 0.53 | 0 - 1.06 | 0.04 |
|  | 25 | 0.53 | 0.000 - 1.056 | 0.04 |
|  | 30 | 0.53 | 0.008 - 1.048 | 0.04 |
|  | 35 | 0.53 | 0.015 - 1.041 | 0.04 |
|  | 40 | 0.53 | -0.03 - 1.087 | 0.04 |
|  | 45 | 0.53 | 0.024 - 1.033 | 0.04 |
|  | 50 | 0.53 | 0 - 1.062 | 0.04 |
|  | 55 | 0.53 | 0.003 - 1.054 | 0.04 |
|  | 60 | 0.53 | -0.01 - 1.076 | 0.04 |
|  | 65 | 0.53 | 0 - 1.06 | 0.04 |
|  | 70 | 0.53 | -0.04 - 1.099 | 0.04 |
|  | 75 | 0.53 | -0.05 - 1.11 | 0.04 |
|  | 80 | 0.53 | 0.005 - 1.052 | 0.04 |
|  | 85 | 0.53 | -0.03 - 1.088 | 0.04 |
|  | 90 | 0.53 | 0 - 1.064 | 0.04 |
|  | 95 | 0.53 | -0.01 - 1.073 | 0.04 |
| 4.7 Capacity to test and approve new medical countermeasures | 15 | -0.53 | -1.04 - -0.02 | 0.53 |
|  | 20 | -0.53 | -1.07 - 0.007 | 0.53 |
|  | 25 | -0.53 | -1.08 - 0.012 | 0.53 |
|  | 30 | -0.10 | -0.63 - 0.418 | 0.23 |
|  | 35 | -0.10 | -0.65 - 0.435 | 0.23 |
|  | 40 | -0.10 | -0.67 - 0.454 | 0.23 |
|  | 45 | -0.10 | -0.64 - 0.431 | 0.23 |
|  | 50 | -0.10 | -0.61 - 0.399 | 0.23 |
|  | 55 | 0.07 | -0.5 - 0.641 | 0.00 |
|  | 60 | 0.07 | -0.49 - 0.628 | 0.00 |
|  | 65 | 0.07 | -0.5 - 0.641 | 0.00 |
|  | 70 | 0.07 | -0.49 - 0.63 | 0.00 |
|  | 75 | 0.07 | -0.52 - 0.659 | 0.00 |
|  | 80 | 0.67 | 0 - 1.345 | 0.00 |
|  | 85 | 0.67 | -0.02 - 1.37 | 0.00 |
|  | 90 | 0.67 | 0 - 1.348 | 0.00 |
|  | 95 | 0.67 | 0.023 - 1.318 | 0.00 |
