# Supplementary material for: Countries’ progress towards Global Health Security (GHS) increased health systems resilience during the Coronavirus Disease-19 (COVID-19) pandemic: A difference-in-difference study of 191 countries
Source: PLOS Glob Public Health. 2025 Jan 7;5(1):e0004051. doi: 10.1371/journal.pgph.0004051 (PMC11706378; doi:10.1371/journal.pgph.0004051)
Supplement: S12 Table — (DOCX) [file pgph.0004051.s014.docx]

**S12 Table. Difference-in-difference model results by year for GHSI Category 4 (Health System) scores which fulfilled the parallel pre-trend assumption at cutoff intervals varying by five (2020-2022).**

| **GHSI Category** | **Cutoff value** | **Average DiD effect size (2020-2022)** | **DiD effect size for 2020** | **DiD effect size for 2021** | **DiD effect size for 2022** | ***p-value* for parallel trend** |
| --- | --- | --- | --- | --- | --- | --- |
| 4.1 Health capacity in clinics, hospitals, and community care centers | 25 | -0.23 (-0.83 - 0.37) | -0.87 (-1.59 - -0.16) | 0.12 (-0.89 - 1.12) | 0.06 (-0.94 - 1.06) | 0.14 |
|  | 30 | 0.46 (-0.07 - 0.98) | -0.21 (-0.88 - 0.45) | 0.65 (-0.32 - 1.61) | 0.94 (0.01 - 1.86) | 0.35 |
|  | 55 | 1.14 (0.59 - 1.69) | 0.85 (0.25 - 1.44) | 1.61 (0.76 - 2.46) | 0.97 (0.09 - 1.86) | 0.22 |
| 4.2 Supply chain for health system and healthcare workers | 75 | 0.86 (0.13 - 1.59) | -0.45 (-1.76 - 0.87) | 1.29 (0.03 - 2.55) | 1.74 (0.82 - 2.66) | 0.33 |
|  | 80 | 0.86 (0.15 - 1.57) | -0.45 (-1.92 - 1.02) | 1.29 (0.06 - 2.52) | 1.74 (0.77 - 2.71) | 0.33 |
| 4.3 Medical countermeasures and personnel deployment | 15 | -0.64 (-1.17 - -0.1) | -0.76 (-1.46 - -0.06) | -1.09 (-2.21 - 0.03) | -0.06 (-0.92 - 0.8) | 0.4 |
|  | 20 | -0.64 (-1.19 - -0.08) | -0.76 (-1.47 - -0.04) | -1.09 (-2.22 - 0.03) | -0.06 (-0.91 - 0.79) | 0.4 |
|  | 25 | -0.64 (-1.18 - -0.1) | -0.76 (-1.47 - -0.04) | -1.09 (-2.22 - 0.04) | -0.06 (-0.9 - 0.78) | 0.4 |
|  | 30 | -0.64 (-1.16 - -0.11) | -0.76 (-1.4 - -0.12) | -1.09 (-2.21 - 0.03) | -0.06 (-0.85 - 0.73) | 0.4 |
|  | 35 | -0.64 (-1.17 - -0.1) | -0.76 (-1.51 - 0) | -1.09 (-2.23 - 0.05) | -0.06 (-0.91 - 0.79) | 0.4 |
|  | 40 | -0.64 (-1.2 - -0.08) | -0.76 (-1.47 - -0.05) | -1.09 (-2.24 - 0.05) | -0.06 (-0.89 - 0.77) | 0.4 |
|  | 45 | -0.64 (-1.17 - -0.1) | -0.76 (-1.47 - -0.05) | -1.09 (-2.18 - 0) | -0.06 (-0.89 - 0.77) | 0.4 |
|  | 50 | -0.64 (-1.14 - -0.14) | -0.76 (-1.4 - -0.11) | -1.09 (-2.18 - -0.01) | -0.06 (-0.9 - 0.78) | 0.4 |
|  | 55 | 2.14 (1.3 - 2.98) | 1.34 (0.22 - 2.47) | 2.9 (1.37 - 4.44) | 2.16 (0.3 - 4.03) | 0.36 |
|  | 60 | 2.14 (1.16 - 3.11) | 1.34 (0.23 - 2.46) | 2.9 (1.38 - 4.43) | 2.16 (0.29 - 4.03) | 0.36 |
|  | 65 | 2.14 (1.25 - 3.02) | 1.34 (0.12 - 2.57) | 2.9 (1.34 - 4.47) | 2.16 (0.25 - 4.07) | 0.36 |
|  | 70 | 2.14 (1.21 - 3.07) | 1.34 (0.19 - 2.5) | 2.9 (1.37 - 4.44) | 2.16 (0.22 - 4.1) | 0.36 |
|  | 75 | 2.14 (1.31 - 2.97) | 1.34 (0.18 - 2.51) | 2.9 (1.3 - 4.51) | 2.16 (0.16 - 4.17) | 0.36 |
|  | 80 | 2.14 (1.26 - 3.01) | 1.34 (0.1 - 2.59) | 2.9 (1.28 - 4.53) | 2.16 (0.36 - 3.96) | 0.36 |
|  | 85 | 2.14 (1.27 - 3) | 1.34 (0.21 - 2.48) | 2.9 (1.4 - 4.41) | 2.16 (0.14 - 4.18) | 0.36 |
|  | 90 | 2.14 (1.29 - 2.98) | 1.34 (0.14 - 2.55) | 2.9 (1.31 - 4.5) | 2.16 (0.21 - 4.11) | 0.36 |
|  | 95 | 2.14 (1.22 - 3.05) | 1.34 (0.26 - 2.43) | 2.9 (1.45 - 4.36) | 2.16 (0.19 - 4.13) | 0.36 |
| 4.4 Healthcare access | 60 | -0.18 (-0.8 - 0.44) | -0.41 (-1.26 - 0.45) | 0.36 (-0.68 - 1.41) | -0.49 (-1.55 - 0.56) | 0.32 |
| 4.5 Communications with healthcare workers during a public health emergency | 15 | 0.28 (-0.39 - 0.96) | -0.44 (-1.33 - 0.45) | 0.77 (-0.59 - 2.14) | 0.52 (-0.46 - 1.5) | 0.2 |
|  | 20 | 0.28 (-0.38 - 0.95) | -0.44 (-1.35 - 0.47) | 0.77 (-0.55 - 2.09) | 0.52 (-0.52 - 1.56) | 0.2 |
|  | 25 | 0.28 (-0.41 - 0.97) | -0.44 (-1.33 - 0.45) | 0.77 (-0.58 - 2.13) | 0.52 (-0.45 - 1.49) | 0.2 |
|  | 30 | 0.28 (-0.39 - 0.96) | -0.44 (-1.33 - 0.45) | 0.77 (-0.66 - 2.21) | 0.52 (-0.51 - 1.54) | 0.2 |
|  | 35 | 0.28 (-0.39 - 0.96) | -0.44 (-1.37 - 0.49) | 0.77 (-0.67 - 2.21) | 0.52 (-0.48 - 1.51) | 0.2 |
|  | 40 | 0.28 (-0.36 - 0.93) | -0.44 (-1.38 - 0.5) | 0.77 (-0.6 - 2.14) | 0.52 (-0.53 - 1.57) | 0.2 |
|  | 45 | 0.28 (-0.37 - 0.93) | -0.44 (-1.32 - 0.44) | 0.77 (-0.65 - 2.2) | 0.52 (-0.56 - 1.6) | 0.2 |
|  | 50 | 0.28 (-0.4 - 0.97) | -0.44 (-1.37 - 0.49) | 0.77 (-0.59 - 2.14) | 0.52 (-0.54 - 1.57) | 0.2 |
| 4.7 Capacity to test and approve new medical countermeasures | 20 | -0.53 (-1.06 - -0.01) | -0.29 (-1.04 - 0.46) | -0.79 (-1.64 - 0.07) | -0.52 (-1.54 - 0.49) | 0.53 |
|  | 25 | -0.53 (-1.04 - -0.03) | -0.29 (-1.07 - 0.49) | -0.79 (-1.67 - 0.09) | -0.52 (-1.53 - 0.48) | 0.53 |
|  | 30 | -0.53 (-1.04 - -0.03) | -0.29 (-1.07 - 0.49) | -0.79 (-1.67 - 0.09) | -0.52 (-1.53 - 0.48) | 0.53 |
|  | 35 | -0.11 (-0.64 - 0.42) | -0.66 (-1.31 - -0.01) | 0.44 (-0.55 - 1.42) | -0.1 (-0.96 - 0.75) | 0.23 |
|  | 40 | -0.11 (-0.61 - 0.39) | -0.66 (-1.33 - 0.01) | 0.44 (-0.67 - 1.55) | -0.1 (-0.98 - 0.77) | 0.23 |
|  | 45 | -0.11 (-0.62 - 0.4) | -0.66 (-1.31 - -0.01) | 0.44 (-0.61 - 1.49) | -0.1 (-1.02 - 0.82) | 0.23 |
|  | 50 | -0.11 (-0.64 - 0.42) | -0.66 (-1.31 - -0.01) | 0.44 (-0.56 - 1.43) | -0.1 (-0.96 - 0.75) | 0.23 |
