# Supplementary material for: Countries’ progress towards Global Health Security (GHS) increased health systems resilience during the Coronavirus Disease-19 (COVID-19) pandemic: A difference-in-difference study of 191 countries
Source: PLOS Glob Public Health. 2025 Jan 7;5(1):e0004051. doi: 10.1371/journal.pgph.0004051 (PMC11706378; doi:10.1371/journal.pgph.0004051)
Supplement: S13 Table — (DOCX) [file pgph.0004051.s015.docx]

**S13 Table. Difference-in-difference model results for GHSI Category 5 (Compliance with International Norms) by cutoff values (2020-2022).**

| **GHSI Category** | **Cutoff Value** | **Average DiD effect size (2020-2022)** | **95% Confidence Interval** | ***p-value* for parallel trend** |
| --- | --- | --- | --- | --- |
| 5.1 International Health Regulations (IHR) reporting compliance and disaster risk reduction | 55 | -0.62 | -1.24 - 0 | 0.45 |
|  | 60 | -0.62 | -1.27 - 0.033 | 0.45 |
|  | 65 | -0.62 | -1.25 - 0.01 | 0.45 |
|  | 70 | -0.62 | -1.26 - 0.021 | 0.45 |
|  | 75 | -0.62 | -1.22 - -0.01 | 0.45 |
|  | 80 | -0.62 | -1.24 - 0 | 0.45 |
|  | 85 | -0.62 | -1.24 - 0.003 | 0.45 |
|  | 90 | -0.62 | -1.26 - 0.023 | 0.45 |
|  | 95 | -0.62 | -1.27 - 0.025 | 0.45 |
| 5.2 Cross-border agreements on public and animal health emergency response | 15 | -0.39 | -1.26 - 0.475 | 0.01 |
|  | 20 | -0.39 | -1.32 - 0.529 | 0.01 |
|  | 25 | -0.39 | -1.23 - 0.439 | 0.01 |
|  | 30 | -0.39 | -1.27 - 0.48 | 0.01 |
|  | 35 | -0.39 | -1.2 - 0.418 | 0.01 |
|  | 40 | -0.39 | -1.27 - 0.486 | 0.01 |
|  | 45 | -0.39 | -1.26 - 0.477 | 0.01 |
|  | 50 | -0.39 | -1.28 - 0.495 | 0.01 |
|  | 55 | 0.80 | 0.023 - 1.576 | 0.00 |
|  | 60 | 0.80 | 0.000 - 1.599 | 0.00 |
|  | 65 | 0.80 | -0.06 - 1.668 | 0.00 |
|  | 70 | 0.80 | 0.033 - 1.567 | 0.00 |
|  | 75 | 0.80 | -0.01 - 1.613 | 0.00 |
|  | 80 | 0.80 | -0.03 - 1.633 | 0.00 |
|  | 85 | 0.80 | -0.01 - 1.617 | 0.00 |
|  | 90 | 0.80 | 0.006 - 1.593 | 0.00 |
|  | 95 | 0.80 | 0 - 1.607 | 0.00 |
| 5.3 International commitments | 30 | -0.48 | -0.92 - -0.03 | 0.01 |
|  | 35 | 0.00 | -0.54 - 0.527 | 0.00 |
|  | 40 | 0.26 | -0.25 - 0.769 | 0.02 |
|  | 45 | -0.03 | -0.53 - 0.458 | 0.00 |
|  | 50 | -0.08 | -0.62 - 0.452 | 0.26 |
|  | 55 | -0.14 | -0.66 - 0.361 | 0.37 |
|  | 60 | -0.14 | -0.67 - 0.37 | 0.37 |
|  | 65 | -0.14 | -0.63 - 0.336 | 0.37 |
|  | 70 | -0.08 | -0.55 - 0.393 | 0.13 |
|  | 75 | -0.08 | -0.57 - 0.404 | 0.13 |
|  | 80 | -0.40 | -0.88 - 0.07 | 0.16 |
|  | 85 | -0.09 | -0.92 - 0.741 | 0.22 |
|  | 90 | -0.18 | -1.09 - 0.732 | 0.46 |
|  | 95 | -0.06 | -1.3 - 1.176 | 0.70 |
| 5.4 Joint External Evaluation (JEE) and Performance of Veterinary Services (PVS) Pathway | 15 | 0.08 | -0.62 - 0.788 | 0.00 |
|  | 20 | 0.08 | -0.6 - 0.773 | 0.00 |
|  | 25 | 0.08 | -0.57 - 0.746 | 0.00 |
|  | 30 | 1.35 | 0.509 - 2.182 | 0.14 |
|  | 35 | 1.35 | 0.573 - 2.118 | 0.14 |
|  | 40 | 1.35 | 0.564 - 2.126 | 0.14 |
|  | 45 | 1.35 | 0.575 - 2.116 | 0.14 |
|  | 50 | 1.35 | 0.579 - 2.112 | 0.14 |
|  | 55 | 0.34 | -0.64 - 1.329 | 0.00 |
|  | 60 | 0.34 | -0.66 - 1.345 | 0.00 |
|  | 65 | 0.34 | -0.67 - 1.354 | 0.00 |
|  | 70 | 0.34 | -0.6 - 1.293 | 0.00 |
|  | 75 | 0.34 | -0.67 - 1.359 | 0.00 |
|  | 80 | -2.46 | -4.66 - -0.25 | 0.00 |
|  | 85 | -2.46 | -4.84 - -0.08 | 0.00 |
|  | 90 | -2.46 | -4.58 - -0.33 | 0.00 |
|  | 95 | -2.46 | -4.68 - -0.23 | 0.00 |
| 5.5 Financing | 35 | -1.76 | -2.36 - -1.16 | 0.00 |
|  | 40 | -1.76 | -2.32 - -1.2 | 0.00 |
|  | 45 | -0.88 | -1.7 - -0.07 | 0.01 |
|  | 50 | -0.96 | -1.79 - -0.13 | 0.01 |
|  | 55 | -0.88 | -1.9 - 0.128 | 0.04 |
|  | 60 | -1.58 | -2.6 - -0.56 | 0.04 |
|  | 65 | -1.62 | -2.68 - -0.56 | 0.01 |
|  | 70 | 0.07 | -0.56 - 0.703 | 0.00 |
|  | 75 | 0.07 | -0.57 - 0.704 | 0.00 |
| 5.6 Commitment to sharing of genetic and biological data and specimens | 70 | 1.75 | 0.382 - 3.124 | 0.04 |
|  | 75 | 1.75 | 0.364 - 3.142 | 0.04 |
|  | 80 | 1.75 | 0.406 - 3.100 | 0.04 |
|  | 85 | 1.75 | 0.413 - 3.093 | 0.04 |
|  | 90 | 1.75 | 0.384 - 3.123 | 0.04 |
|  | 95 | 1.75 | 0.348 - 3.159 | 0.04 |
