# Supplementary material for: Countries’ progress towards Global Health Security (GHS) increased health systems resilience during the Coronavirus Disease-19 (COVID-19) pandemic: A difference-in-difference study of 191 countries
Source: PLOS Glob Public Health. 2025 Jan 7;5(1):e0004051. doi: 10.1371/journal.pgph.0004051 (PMC11706378; doi:10.1371/journal.pgph.0004051)
Supplement: S14 Table — (DOCX) [file pgph.0004051.s016.docx]

**S14 Table. Difference-in-difference model results by year for GHSI Category 5 (Compliance with International Norms) scores which fulfilled the parallel pre-trend assumption at cutoff intervals varying by five (2020-2022).**

| **GHSI Category** | **Cutoff value** | **Average DiD effect size (2020-2022)** | **DiD effect size for 2020** | **DiD effect size for 2021** | **DiD effect size for 2022** | ***p-value* for parallel trend** |
| --- | --- | --- | --- | --- | --- | --- |
| 5.1 International Health Regulations (IHR) reporting compliance and disaster risk reduction | 55 | -0.62 (-1.26 - 0.02) | -0.3 (-1.12 - 0.52) | -0.93 (-2.23 - 0.38) | -0.64 (-1.69 - 0.41) | 0.45 |
|  | 60 | -0.62 (-1.29 - 0.04) | -0.3 (-1.13 - 0.53) | -0.93 (-2.2 - 0.35) | -0.64 (-1.67 - 0.39) | 0.45 |
|  | 65 | -0.62 (-1.27 - 0.03) | -0.3 (-1.11 - 0.51) | -0.93 (-2.21 - 0.35) | -0.64 (-1.66 - 0.39) | 0.45 |
|  | 70 | -0.62 (-1.24 - 0) | -0.3 (-1.1 - 0.5) | -0.93 (-2.17 - 0.32) | -0.64 (-1.59 - 0.32) | 0.45 |
|  | 75 | -0.62 (-1.3 - 0.06) | -0.3 (-1.15 - 0.55) | -0.93 (-2.22 - 0.36) | -0.64 (-1.66 - 0.38) | 0.45 |
|  | 80 | -0.62 (-1.23 - -0.02) | -0.3 (-1.11 - 0.51) | -0.93 (-2.26 - 0.4) | -0.64 (-1.65 - 0.38) | 0.45 |
|  | 85 | -0.62 (-1.25 - 0) | -0.3 (-1.13 - 0.53) | -0.93 (-2.23 - 0.37) | -0.64 (-1.67 - 0.39) | 0.45 |
|  | 90 | -0.62 (-1.28 - 0.03) | -0.3 (-1.12 - 0.52) | -0.93 (-2.19 - 0.33) | -0.64 (-1.66 - 0.38) | 0.45 |
|  | 95 | -0.62 (-1.27 - 0.02) | -0.3 (-1.13 - 0.52) | -0.93 (-2.17 - 0.31) | -0.64 (-1.63 - 0.36) | 0.45 |
| 5.3 International commitments | 50 | -0.09 (-0.57 - 0.39) | -0.04 (-0.72 - 0.63) | 0.28 (-0.53 - 1.09) | -0.5 (-1.28 - 0.28) | 0.26 |
|  | 55 | -0.15 (-0.62 - 0.32) | 0 (-0.65 - 0.65) | 0.18 (-0.67 - 1.03) | -0.63 (-1.41 - 0.16) | 0.37 |
|  | 60 | -0.15 (-0.64 - 0.34) | 0 (-0.66 - 0.66) | 0.18 (-0.69 - 1.04) | -0.63 (-1.43 - 0.18) | 0.37 |
|  | 65 | -0.15 (-0.62 - 0.32) | 0 (-0.71 - 0.7) | 0.18 (-0.74 - 1.09) | -0.63 (-1.42 - 0.17) | 0.37 |
|  | 70 | -0.08 (-0.54 - 0.37) | -0.01 (-0.64 - 0.62) | 0.29 (-0.62 - 1.2) | -0.53 (-1.3 - 0.25) | 0.13 |
|  | 75 | -0.08 (-0.52 - 0.36) | -0.01 (-0.6 - 0.58) | 0.29 (-0.54 - 1.12) | -0.53 (-1.32 - 0.26) | 0.13 |
|  | 80 | -0.41 (-0.87 - 0.06) | -0.25 (-0.95 - 0.44) | 0.05 (-0.87 - 0.96) | -1.02 (-1.89 - -0.14) | 0.16 |
|  | 85 | -0.09 (-1 - 0.81) | -0.4 (-1.14 - 0.33) | 0.88 (-1.87 - 3.63) | -0.76 (-1.68 - 0.17) | 0.22 |
|  | 90 | -0.18 (-1.08 - 0.71) | -0.68 (-1.39 - 0.03) | 1.01 (-1.99 - 4.02) | -0.89 (-1.79 - 0.02) | 0.46 |
|  | 95 | -0.06 (-1.25 - 1.13) | -0.22 (-0.89 - 0.44) | 0.8 (-3.17 - 4.76) | -0.76 (-1.75 - 0.22) | 0.7 |
| 5.4 Joint External Evaluation (JEE) and Performance of Veterinary Services (PVS) Pathway | 30 | 1.35 (0.54 - 2.15) | 1 (0.21 - 1.78) | 0.89 (-0.82 - 2.6) | 2.15 (0.61 - 3.69) | 0.14 |
|  | 35 | 1.35 (0.55 - 2.14) | 1 (0.22 - 1.77) | 0.89 (-0.74 - 2.52) | 2.15 (0.62 - 3.68) | 0.14 |
|  | 40 | 1.35 (0.59 - 2.1) | 1 (0.19 - 1.8) | 0.89 (-0.83 - 2.62) | 2.15 (0.67 - 3.63) | 0.14 |
|  | 45 | 1.35 (0.59 - 2.1) | 1 (0.2 - 1.79) | 0.89 (-0.85 - 2.63) | 2.15 (0.67 - 3.63) | 0.14 |
|  | 50 | 1.35 (0.54 - 2.15) | 1 (0.25 - 1.75) | 0.89 (-0.85 - 2.64) | 2.15 (0.6 - 3.7) | 0.14 |
