# Supplementary material for: Countries’ progress towards Global Health Security (GHS) increased health systems resilience during the Coronavirus Disease-19 (COVID-19) pandemic: A difference-in-difference study of 191 countries
Source: PLOS Glob Public Health. 2025 Jan 7;5(1):e0004051. doi: 10.1371/journal.pgph.0004051 (PMC11706378; doi:10.1371/journal.pgph.0004051)
Supplement: S15 Table — (DOCX) [file pgph.0004051.s017.docx]

**S15 Table. Difference-in-difference model results for GHSI Category 6 (Risk Environment) by cutoff values (2020-2022).**

| **GHSI Indicator** | **Cutoff Value** | **Average DiD effect size (2020-2022)** | **95% Confidence Interval** | ***p-value* for parallel trend** |
| --- | --- | --- | --- | --- |
| 6.1 Political and security risk | 45 | -0.38 | -1.58 - 0.819 | 0.01 |
|  | 50 | -1.26 | -2.26 - -0.26 | 0.01 |
|  | 55 | -2.11 | -3.02 - -1.2 | 0.00 |
|  | 60 | -0.28 | -1.29 - 0.722 | 0.00 |
|  | 65 | 0.06 | -0.81 - 0.938 | 0.00 |
|  | 70 | 0.33 | -0.31 - 0.973 | 0.00 |
|  | 75 | 0.29 | -0.34 - 0.92 | 0.00 |
|  | 80 | -0.22 | -0.94 - 0.491 | 0.18 |
|  | 85 | 0.91 | 0.143 - 1.672 | 0.00 |
|  | 90 | 0.17 | -1.71 - 2.057 | 0.01 |
|  | 95 | 3.91 | 3.286 - 4.529 | 0.00 |
| 6.2 Socioeconomic resilience | 55 | -2.03 | -3.2 - -0.86 | 0.00 |
|  | 60 | -1.59 | -2.5 - -0.68 | 0.02 |
|  | 65 | -1.54 | -2.26 - -0.83 | 0.05 |
|  | 70 | -0.37 | -0.92 - 0.182 | 0.00 |
|  | 75 | 0.22 | -0.36 - 0.802 | 0.07 |
|  | 80 | 0.42 | -0.2 - 1.046 | 0.02 |
|  | 85 | 0.37 | -0.25 - 0.998 | 0.06 |
|  | 90 | 0.19 | -0.51 - 0.905 | 0.99 |
|  | 95 | 0.61 | 0.070 - 1.146 | 0.21 |
| 6.3 Infrastructure adequacy | 35 | -0.30 | -1.58 - 0.967 | 0.00 |
|  | 40 | -0.30 | -1.53 - 0.918 | 0.00 |
|  | 45 | -0.51 | -1.6 - 0.585 | 0.00 |
|  | 50 | -0.51 | -1.58 - 0.565 | 0.00 |
|  | 55 | -0.46 | -1.2 - 0.27 | 0.00 |
|  | 60 | -0.52 | -1.17 - 0.112 | 0.43 |
|  | 65 | -0.52 | -1.13 - 0.075 | 0.43 |
|  | 70 | 0.56 | -0.11 - 1.241 | 0.04 |
|  | 75 | 0.56 | -0.16 - 1.286 | 0.04 |
|  | 80 | 0.80 | 0.228 - 1.363 | 0.12 |
|  | 85 | 0.96 | 0.469 - 1.446 | 0.81 |
|  | 90 | 0.96 | 0.416 - 1.500 | 0.81 |
|  | 95 | 1.17 | 0.773 - 1.567 | 0.00 |
| 6.4 Environmental Risks | 45 | 1.17 | 0.352 - 1.994 | 0.00 |
|  | 50 | 1.27 | 0.516 - 2.021 | 0.00 |
|  | 55 | 1.32 | 0.678 - 1.956 | 0.00 |
|  | 60 | 2.13 | 1.611 - 2.657 | 0.00 |
|  | 65 | 2.78 | 2.097 - 3.455 | 0.00 |
|  | 70 | 2.16 | 1.044 - 3.271 | 0.00 |
|  | 75 | 3.35 | 1.570 - 5.124 | 0.00 |
|  | 80 | 0.99 | -0.75 - 2.731 | 0.21 |
| 6.5 Public health vulnerabilities | 50 | 1.57 | 0.421 - 2.709 | 0.00 |
|  | 55 | 0.20 | -0.5 - 0.901 | 0.01 |
|  | 60 | 1.41 | 0.838 - 1.991 | 0.00 |
|  | 65 | 0.99 | 0.506 - 1.464 | 0.01 |
|  | 70 | 1.07 | 0.473 - 1.669 | 0.01 |
|  | 75 | 0.67 | 0.121 - 1.215 | 0.01 |
|  | 80 | 0.56 | 0.139 - 0.976 | 0.00 |
|  | 85 | 0.93 | 0.529 - 1.335 | 0.73 |
|  | 90 | 0.82 | 0.427 - 1.216 | 0.03 |
