# Supplementary material for: Countries’ progress towards Global Health Security (GHS) increased health systems resilience during the Coronavirus Disease-19 (COVID-19) pandemic: A difference-in-difference study of 191 countries
Source: PLOS Glob Public Health. 2025 Jan 7;5(1):e0004051. doi: 10.1371/journal.pgph.0004051 (PMC11706378; doi:10.1371/journal.pgph.0004051)
Supplement: S16 Table — (DOCX) [file pgph.0004051.s018.docx]

**S16 Table. Difference-in-difference model results by year for GHSI Category 6 (Risk Environment) scores which fulfilled the parallel pre-trend assumption at cutoff intervals varying by five (2020-2022).**

| **GHSI Category** | **Cutoff value** | **Average DiD effect size (2020-2022)** | **DiD effect size for 2020** | **DiD effect size for 2021** | **DiD effect size for 2022** | ***p-value* for parallel trend** |
| --- | --- | --- | --- | --- | --- | --- |
| 6.1 Political and security risk | 80 | -0.23 (-0.96 - 0.5) | 0.07 (-0.58 - 0.71) | -0.58 (-2.43 - 1.26) | -0.17 (-1.14 - 0.81) | 0.18 |
| 6.2 Socioeconomic resilience | 90 | 0.10 (-0.67 - 0.88) | 0.55 (0.08 - 1.01) | 0.21 (-2.38 - 2.8) | -0.45 (-1.43 - 0.52) | 0.80 |
|  | 95 | 0.61 (0.09 - 1.13) | 0.48 (-0.01 - 0.96) | 1.48 (0.16 - 2.81) | -0.13 (-1.18 - 0.92) | 0.21 |
| 6.3 Infrastructure adequacy | 60 | -0.53 (-1.14 - 0.08) | -0.3 (-1.04 - 0.43) | -0.34 (-1.39 - 0.72) | -0.95 (-1.87 - -0.02) | 0.43 |
|  | 65 | -0.53 (-1.07 - 0.01) | -0.3 (-1 - 0.4) | -0.34 (-1.25 - 0.58) | -0.95 (-1.82 - -0.07) | 0.43 |
|  | 80 | 0.80 (0.27 - 1.33) | 0.64 (0.01 - 1.26) | 0.89 (-0.55 - 2.34) | 0.86 (0.1 - 1.62) | 0.12 |
|  | 85 | 0.96 (0.42 - 1.5) | 0.08 (-0.65 - 0.8) | 1.44 (0.19 - 2.7) | 1.36 (0.49 - 2.22) | 0.81 |
|  | 90 | 0.96 (0.41 - 1.51) | 0.08 (-0.63 - 0.78) | 1.44 (0.22 - 2.66) | 1.36 (0.53 - 2.19) | 0.81 |
| 6.4 Environmental Risks | 80 | 0.99 (-0.76 - 2.73) | 0.12 (-2.74 - 2.97) | 3.02 (0.53 - 5.52) | -0.18 (-3.16 - 2.8) | 0.21 |
| 6.5 Public health vulnerabilities | 85 | 0.93 (0.53 - 1.34) | 0.27 (-0.09 - 0.62) | 1.32 (0.14 - 2.51) | 1.21 (0.52 - 1.89) | 0.73 |
