# Supplementary material for: Countries’ progress towards Global Health Security (GHS) increased health systems resilience during the Coronavirus Disease-19 (COVID-19) pandemic: A difference-in-difference study of 191 countries
Source: PLOS Glob Public Health. 2025 Jan 7;5(1):e0004051. doi: 10.1371/journal.pgph.0004051 (PMC11706378; doi:10.1371/journal.pgph.0004051)
Supplement: S17 Table — (DOCX) [file pgph.0004051.s019.docx]

**S17 Table. Difference-in-difference model results for World Bank Governance Indexes (Effective Governance, Rule of Law, Control of Corruption) by cutoff values (2020-2022).**

| **GHSI Indicator** | **Cutoff Value** | **Average DiD effect size (2020-2022)** | **95% Confidence Interval** | ***p-value* for parallel trend** |
| --- | --- | --- | --- | --- |
| 1. Governance Effectiveness | 0.1 | 0.34 | -0.4 - 1.072 | 0.05 |
|  | 0.2 | 0.49 | -0.34 - 1.32 | 0.02 |
|  | 0.3 | 0.61 | -0.17 - 1.388 | 0.00 |
|  | 0.4 | 0.06 | -0.71 - 0.835 | 0.00 |
|  | 0.5 | 0.26 | -0.35 - 0.869 | 0.16 |
|  | 0.6 | 0.28 | -0.4 - 0.963 | 0.23 |
|  | 0.7 | 0.48 | -0.2 - 1.162 | 0.09 |
|  | 0.8 | 0.48 | -0.19 - 1.148 | 0.09 |
|  | 0.9 | 0.69 | 0.013 - 1.363 | 0.01 |
|  | 1 | 0.47 | -0.17 - 1.102 | 0.00 |
|  | 1.1 | 0.80 | 0.236 - 1.372 | 0.00 |
|  | 1.2 | 0.84 | 0.228 - 1.454 | 0.00 |
|  | 1.3 | 0.80 | 0.166 - 1.442 | 0.00 |
|  | 1.4 | 0.85 | 0.197 - 1.493 | 0.03 |
|  | 1.5 | 0.56 | -0.16 - 1.275 | 0.00 |
|  | 1.6 | 0.80 | 0.331 - 1.263 | 0.02 |
|  | 1.7 | 1.04 | 0.568 - 1.503 | 0.09 |
|  | 1.8 | 0.87 | 0.373 - 1.365 | 0.06 |
|  | 1.9 | 1.02 | 0.439 - 1.605 | 0.03 |
| 2. Rule of Law | 0 | -0.44 | -1.28 - 0.407 | 0.00 |
|  | 0.1 | -0.54 | -1.35 - 0.271 | 0.00 |
|  | 0.2 | -0.37 | -1.08 - 0.343 | 0.02 |
|  | 0.3 | -0.18 | -0.78 - 0.421 | 0.04 |
|  | 0.4 | 0.48 | -0.24 - 1.21 | 0.03 |
|  | 0.5 | 0.63 | -0.05 - 1.32 | 0.04 |
|  | 0.6 | 0.47 | -0.29 - 1.225 | 0.00 |
|  | 0.7 | 0.31 | -0.41 - 1.043 | 0.00 |
|  | 0.8 | 0.63 | -0.03 - 1.298 | 0.00 |
|  | 0.9 | 0.79 | 0.046 - 1.541 | 0.00 |
|  | 1 | 0.71 | 0 - 1.416 | 0.00 |
|  | 1.1 | 0.78 | 0.197 - 1.357 | 0.02 |
|  | 1.2 | 0.87 | 0.272 - 1.463 | 0.08 |
|  | 1.3 | 1.08 | 0.507 - 1.642 | 0.07 |
|  | 1.4 | 0.96 | 0.302 - 1.611 | 0.03 |
|  | 1.5 | 0.62 | 0.014 - 1.222 | 0.01 |
|  | 1.6 | 0.63 | -0.05 - 1.314 | 0.00 |
|  | 1.7 | 0.46 | -0.32 - 1.248 | 0.00 |
|  | 1.8 | 0.67 | 0.168 - 1.169 | 0.00 |
|  | 1.9 | 0.90 | 0.112 - 1.679 | 0.20 |
| 3. Control of Corruption | -0.1 | 0.58 | -0.19 - 1.364 | 0.01 |
|  | 0 | 0.37 | -0.48 - 1.215 | 0.00 |
|  | 0.1 | 0.20 | -0.49 - 0.891 | 0.00 |
|  | 0.2 | 0.42 | -0.32 - 1.165 | 0.00 |
|  | 0.3 | 0.38 | -0.23 - 0.988 | 0.00 |
|  | 0.4 | 0.19 | -0.35 - 0.74 | 0.00 |
|  | 0.5 | 0.20 | -0.4 - 0.809 | 0.00 |
|  | 0.6 | 0.35 | -0.26 - 0.952 | 0.00 |
|  | 0.7 | 0.67 | 0.070 - 1.269 | 0.00 |
|  | 0.8 | 0.72 | 0.084 - 1.360 | 0.00 |
|  | 0.9 | 0.52 | -0.14 - 1.193 | 0.00 |
|  | 1 | 0.50 | -0.16 - 1.162 | 0.00 |
|  | 1.1 | 0.60 | -0.03 - 1.228 | 0.00 |
|  | 1.2 | 1.17 | 0.547 - 1.784 | 0.01 |
|  | 1.3 | 0.85 | 0.219 - 1.476 | 0.01 |
|  | 1.4 | 0.85 | 0.263 - 1.431 | 0.01 |
|  | 1.5 | 0.76 | 0.025 - 1.491 | 0.01 |
|  | 1.6 | 0.92 | 0.150 - 1.689 | 0.03 |
|  | 1.7 | 0.51 | -0.23 - 1.263 | 0.02 |
|  | 1.8 | 0.65 | 0.191 - 1.112 | 0.01 |
|  | 1.9 | 0.58 | 0.090 - 1.061 | 0.04 |
|  | 2 | 0.36 | -0.1 - 0.833 | 0.02 |
