# Supplementary material for: Countries’ progress towards Global Health Security (GHS) increased health systems resilience during the Coronavirus Disease-19 (COVID-19) pandemic: A difference-in-difference study of 191 countries
Source: PLOS Glob Public Health. 2025 Jan 7;5(1):e0004051. doi: 10.1371/journal.pgph.0004051 (PMC11706378; doi:10.1371/journal.pgph.0004051)
Supplement: S18 Table — (DOCX) [file pgph.0004051.s020.docx]

**S18 Table. Difference-in-difference model results by year for World Bank Governance Index (Effective Governance, Rule of Law, Control of Corruption) scores which fulfilled the parallel pre-trend assumption at cutoff intervals varying by five (2020-2022).**

| **GHSI Category** | **Cutoff value** | **Average DiD effect size (2020-2022)** | **DiD effect size for 2020** | **DiD effect size for 2021** | **DiD effect size for 2022** | ***p-value* for parallel trend** |
| --- | --- | --- | --- | --- | --- | --- |
| 1. Governance Effectiveness | 0.5 | 0.26 (-0.35 - 0.87) | 0.88 (-0.05 - 1.8) | -0.14 (-1.21 - 0.92) | 0.04 (-0.92 - 1) | 0.16 |
|  | 0.6 | 0.28 (-0.38 - 0.94) | 0.52 (-0.36 - 1.41) | 0.07 (-0.97 - 1.12) | 0.23 (-0.77 - 1.23) | 0.23 |
| 2. Rule of Law | 1.9 | 0.90 (0.17 - 1.63) | 0.39 (-0.38 - 1.16) | 0.75 (-0.75 - 2.25) | 1.55 (0.44 - 2.67) | 0.20 |
