# Supplementary figures and images for: Countries’ progress towards Global Health Security (GHS) increased health systems resilience during the Coronavirus Disease-19 (COVID-19) pandemic: A difference-in-difference study of 191 countries
Source: PLOS Glob Public Health. 2025 Jan 7;5(1):e0004051. doi: 10.1371/journal.pgph.0004051 (PMC11706378; doi:10.1371/journal.pgph.0004051)

**S1 Fig. Distribution of Original and Recomputed Overall Global Health Security Index (2019) Scores.**


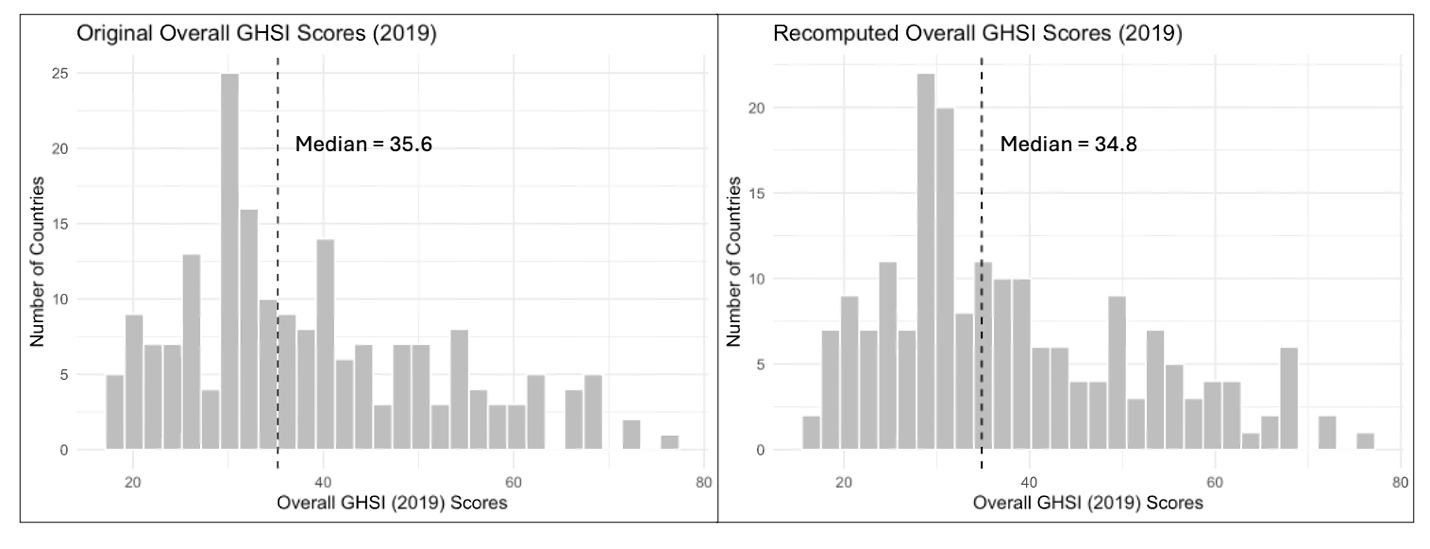

Supplement: S1 Fig — (DOCX) [file pgph.0004051.s021.docx]

**S3 Fig. Distribution of Original and Recomputed Category 1 (Prevention) Scores, 2019.**

**
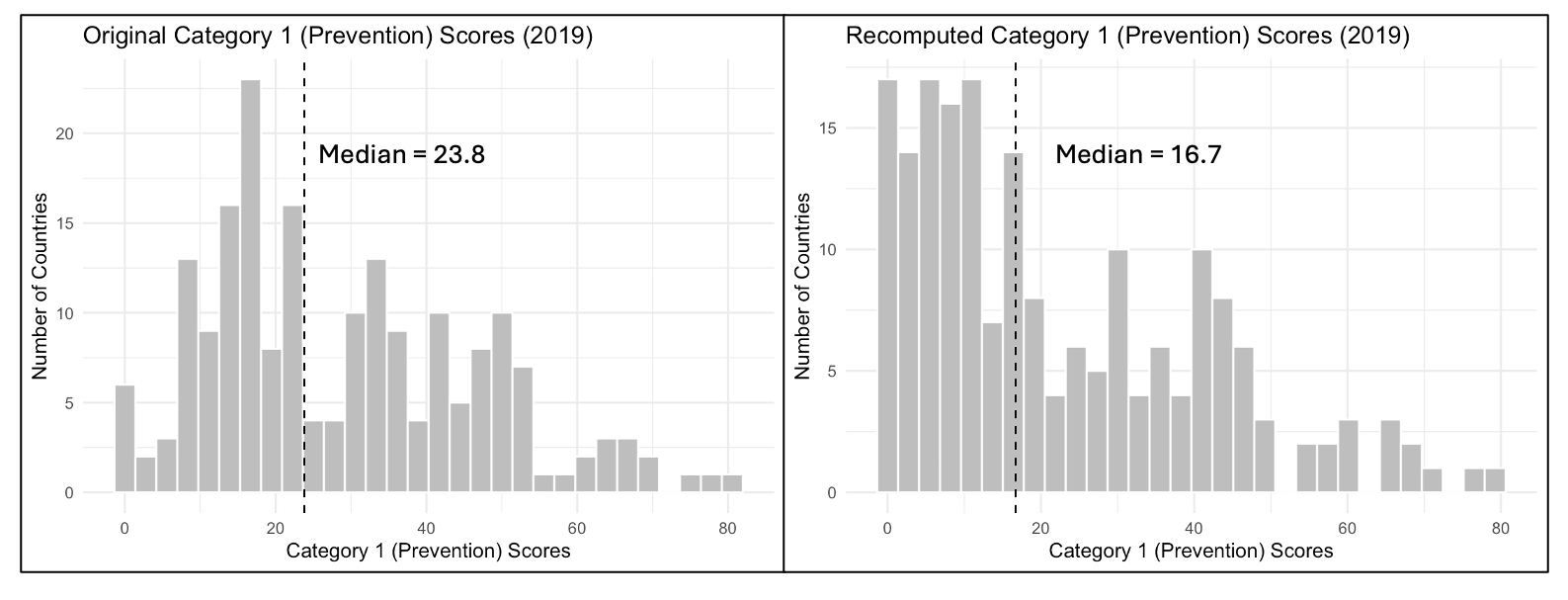
**

Supplement: S3 Fig — (DOCX) [file pgph.0004051.s023.docx]
