# Supplementary material for: Countries’ progress towards Global Health Security (GHS) increased health systems resilience during the Coronavirus Disease-19 (COVID-19) pandemic: A difference-in-difference study of 191 countries
Source: PLOS Glob Public Health. 2025 Jan 7;5(1):e0004051. doi: 10.1371/journal.pgph.0004051 (PMC11706378; doi:10.1371/journal.pgph.0004051)
Supplement: S2 Fig — (DOCX) [file pgph.0004051.s022.docx]

**S2 Fig. Parallel Pre-Trends Between Treatment and Control Groups for Overall GHSI Scores and GHSI Categories (2015-2019).**

**
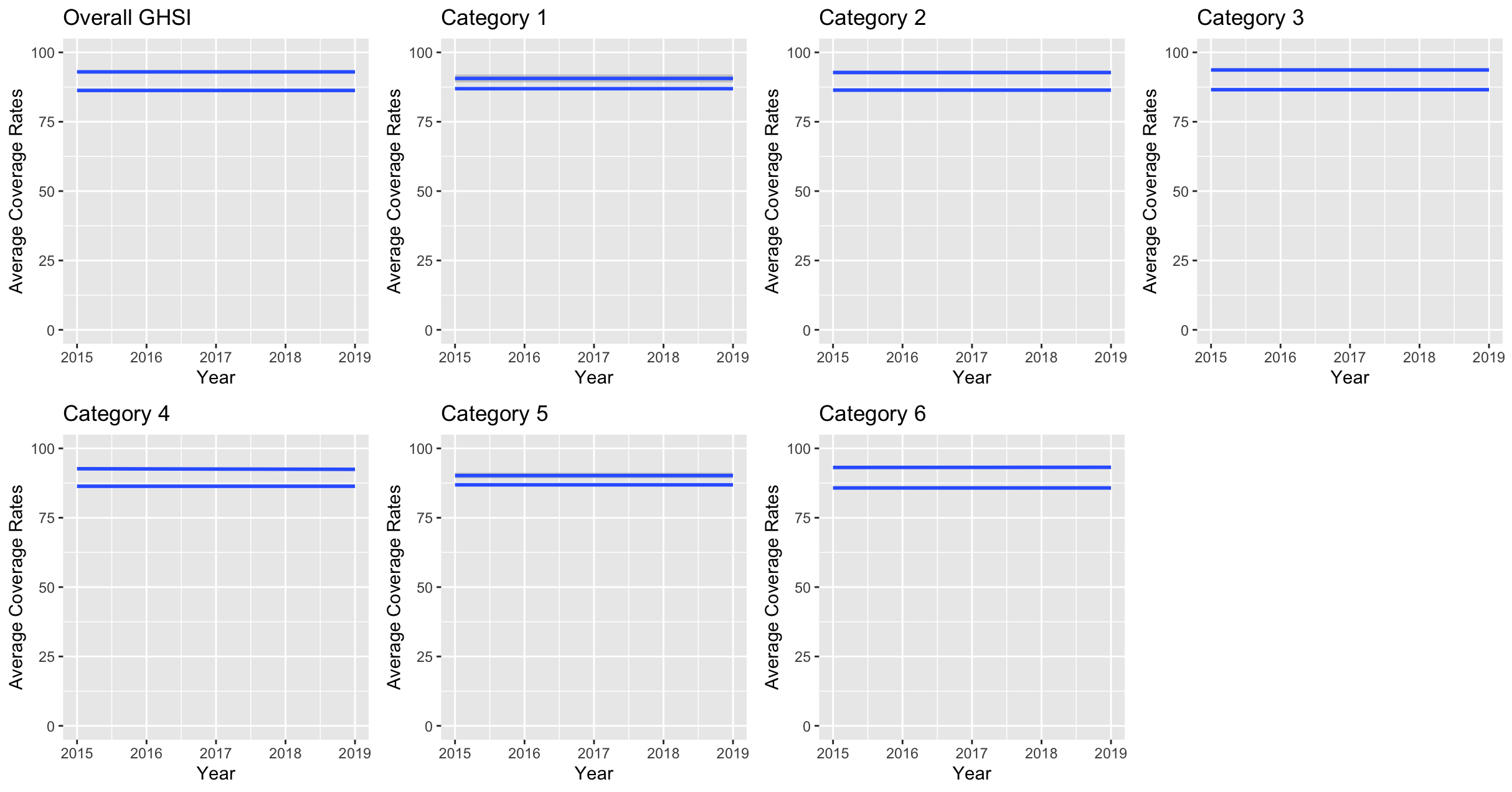
**

**The two lines represent the treatment (upper line) and control (lower line) groups. The average coverage rate refers to the average vaccination rate per year per group (i.e., treatment and control).*
