# Supplementary material for: Countries’ progress towards Global Health Security (GHS) increased health systems resilience during the Coronavirus Disease-19 (COVID-19) pandemic: A difference-in-difference study of 191 countries
Source: PLOS Glob Public Health. 2025 Jan 7;5(1):e0004051. doi: 10.1371/journal.pgph.0004051 (PMC11706378; doi:10.1371/journal.pgph.0004051)
Supplement: S9 Fig — (DOCX) [file pgph.0004051.s029.docx]

**S9 Fig. Difference-in-difference model results for GHSI Category 6 (Risk Environment) (2020-2022)**.

**
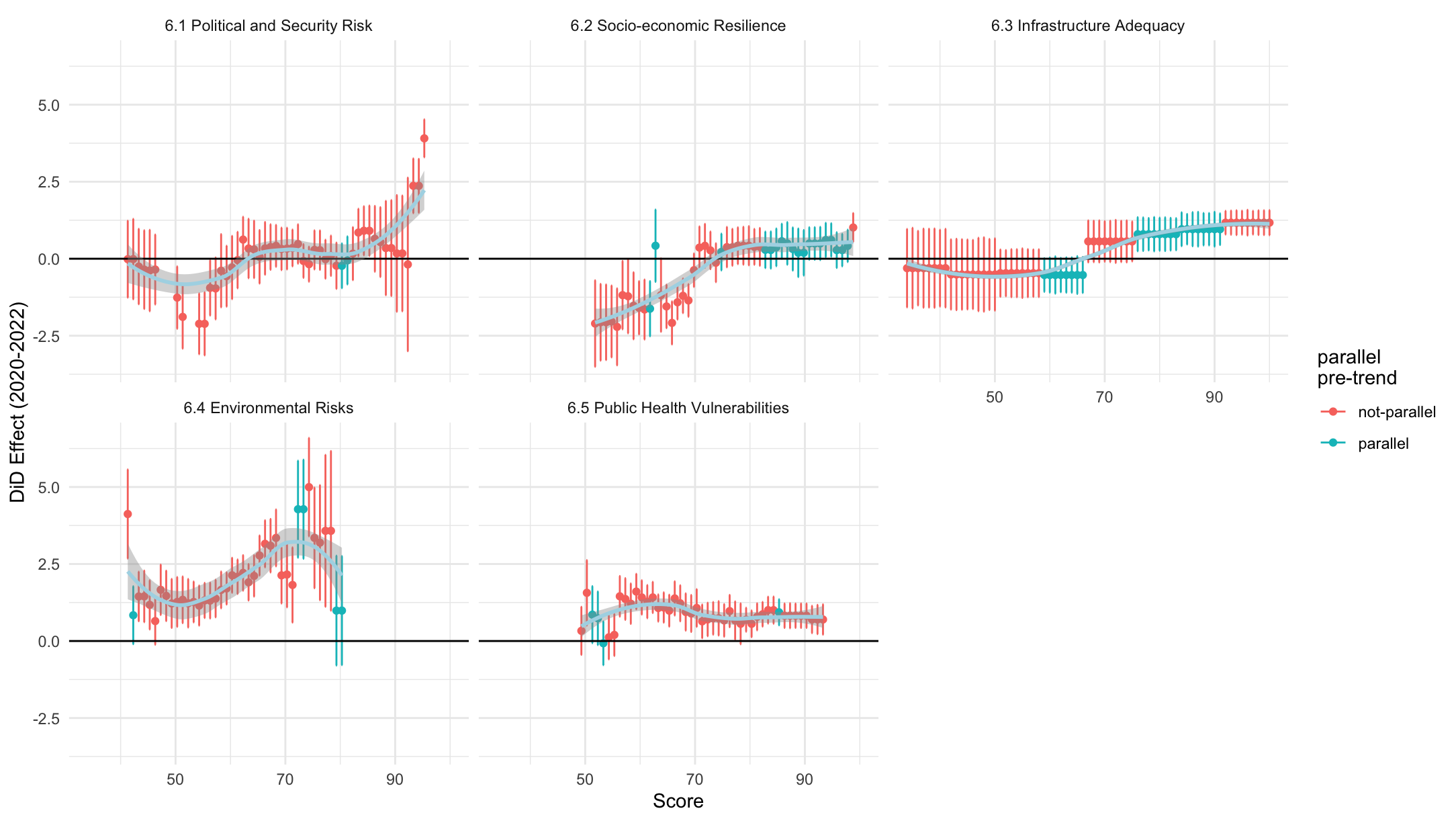
**

*Note: DiD models were only run when there were pre-treatment periods to test. Trendlines and their standard errors (in light blue) were calculated based on local polynomial regression fitting.*
