# Supplementary material for: Countries’ progress towards Global Health Security (GHS) increased health systems resilience during the Coronavirus Disease-19 (COVID-19) pandemic: A difference-in-difference study of 191 countries
Source: PLOS Glob Public Health. 2025 Jan 7;5(1):e0004051. doi: 10.1371/journal.pgph.0004051 (PMC11706378; doi:10.1371/journal.pgph.0004051)
Supplement: Attachment — Submitted filename: GHSI Response to Reviewers.docx [file pgph.0004051.s031.docx]

October 15, 2024

Dear Dr. Mukherjee,

Thank you for your thoughtful comments and for the opportunity to revise our manuscript titled “Countries’ Progress Towards Global Health Security (GHS) Increased Health Systems Resilience During the Coronavirus Disease-19 (COVID-19) Pandemic: A Difference-in-Difference Study of 191 Countries” (**PGPH-D-24-01417**). We greatly appreciate the time and effort that both you and the reviewers have dedicated to providing constructive feedback. The revisions have significantly improved the quality and clarity of our work, and we believe the manuscript is now much stronger.

We have addressed all the reviewer comments point by point, and the manuscript has been updated accordingly. A marked-up version with track changes has been provided for your review, along with a clean version of the revised manuscript. In our point-by-point response, we have outlined the changes made. The most significant edits we made based on the reviewers’ comments were: (1) adding two new figures to provide more context for our methodology and findings; (2) editing our Methods section to clarify our empirical strategy, including adding a more detailed explanation of our difference-in-difference model; and (3) drawing from the literature to explore potential mechanisms driving our findings.

We hope that the revised manuscript meets your expectations and the reviewers' concerns. Please do not hesitate to contact us if further clarifications are needed.

Thank you again for your support and consideration.

Sincerely,

Yesim Tozan
Associate Professor of Global Health

tozan@nyu.edu

1. We have noticed that you have uploaded Supporting Information files, but you have not included **a list of legends**. Please add a full list of legends for your Supporting Information files after the references list. >> We have added a full list of legends to our main text and broke our Supporting Information file (which originally was one file with all of our figures, tables, etc.) into individual files.

2. We note that your Data Availability Statement is currently as follows: "All data used in this analysis is publicly available and citations to the dataset are available through the reference list." >> We have added our data and code to a publicly available repository and included a URL in the main text.
 
 Please confirm at this time whether or not your submission contains all raw data required to replicate the results of your study. Authors must share the “minimal data set” for their submission. PLOS defines the minimal data set to consist of the data required to replicate all study findings reported in the article, as well as related metadata and methods ([https://journals.plos.org/plosone/s/data-availability#loc-minimal-data-set-definition](https://urldefense.proofpoint.com/v2/url?u=https-3A__journals.plos.org_plosone_s_data-2Davailability-23loc-2Dminimal-2Ddata-2Dset-2Ddefinition&d=DwQFaQ&c=slrrB7dE8n7gBJbeO0g-IQ&r=XGzUN6S7CV-sOjf7f4TPQQ&m=ZYgo-n3jro_kZjbpyVQvNmNympc-4C61WWQ_tJauGbWwf3CZ1e2EvPVeZxpfz9GE&s=9k_8Vs1OJ0D1se_AsWK-pqjanPn-4Lq-TErHnBOJqRU&e=)). >>We can confirm that this data is available in the aforementioned publicly available repository.
 
 For example, authors should submit the following data:
 
 - The values behind the means, standard deviations and other measures reported;
 - The values used to build graphs;
 - The points extracted from images for analysis.
 
 Authors do not need to submit their entire data set if only a portion of the data was used in the reported study.
 
 **If your submission does not contain these data, please either upload them as Supporting Information files or deposit them to a stable, public repository and provide us with the relevant URLs, DOIs, or accession numbers**. For a list of recommended repositories, please see [https://journals.plos.org/plosone/s/recommended-repositories](https://urldefense.proofpoint.com/v2/url?u=https-3A__journals.plos.org_plosone_s_recommended-2Drepositories&d=DwQFaQ&c=slrrB7dE8n7gBJbeO0g-IQ&r=XGzUN6S7CV-sOjf7f4TPQQ&m=ZYgo-n3jro_kZjbpyVQvNmNympc-4C61WWQ_tJauGbWwf3CZ1e2EvPVeZxpfz9GE&s=uI8eICOUDUCNy5MG2pt5wkPqBecfYUyqyVJhgNW6ZZc&e=).
 
 If there are ethical or legal restrictions on sharing a de-identified data set, please explain them in detail (e.g., data contain potentially sensitive information, data are owned by a third-party organization, etc.) and who has imposed them (e.g., an ethics committee). Please also provide contact information for a data access committee, ethics committee, or other institutional body to which data requests may be sent. If data are owned by a third party, please indicate how others may request data access.

Please review your reference list to ensure that it is complete and correct. If you have cited papers that have been retracted, please include the rationale for doing so in the manuscript text, or remove these references and replace them with relevant current references. Any changes to the reference list should be mentioned in the rebuttal letter that accompanies your revised manuscript. If you need to cite a retracted article, indicate the article’s retracted status in the References list and also include a citation and full reference for the retraction notice.

Additional Editor Comments (if provided):


[Note: HTML markup is below. Please do not edit.]

Reviewers' comments:

Reviewer's Responses to Questions

**Comments to the Author**

1. Does this manuscript meet PLOS Global Public Health’s [publication criteria](https://urldefense.proofpoint.com/v2/url?u=https-3A__c05y1x9s.r.us-2Deast-2D2.awstrack.me_L0_https-3A-252F-252Fjournals.plos.org-252Fglobalpublichealth-252Fs-252Fcriteria-2Dfor-2Dpublication_1_010f01920eee22e6-2D8cff98ad-2De74f-2D4f8d-2Dabdd-2Df7d752fe646c-2D000000_ygcqVHBPIXgcI3CAUi6la6M1Fso-3D177&d=DwMFaQ&c=slrrB7dE8n7gBJbeO0g-IQ&r=XGzUN6S7CV-sOjf7f4TPQQ&m=ZYgo-n3jro_kZjbpyVQvNmNympc-4C61WWQ_tJauGbWwf3CZ1e2EvPVeZxpfz9GE&s=B5ZxhiTtlVbQ2xNdIc7824JAavrp1tMRnm0CQoSvtJc&e=)? Is the manuscript technically sound, and do the data support the conclusions? The manuscript must describe methodologically and ethically rigorous research with conclusions that are appropriately drawn based on the data presented.

Reviewer #1: Yes

Reviewer #2: Yes

2. Has the statistical analysis been performed appropriately and rigorously?

Reviewer #1: Yes

Reviewer #2: I don't know

3. Have the authors made all data underlying the findings in their manuscript fully available (please refer to the Data Availability Statement at the start of the manuscript PDF file)?

The [PLOS Data policy](https://urldefense.proofpoint.com/v2/url?u=https-3A__c05y1x9s.r.us-2Deast-2D2.awstrack.me_L0_https-3A-252F-252Fjournals.plos.org-252Fglobalpublichealth-252Fs-252Fdata-2Davailability_1_010f01920eee22e6-2D8cff98ad-2De74f-2D4f8d-2Dabdd-2Df7d752fe646c-2D000000_jeL5UYwJP1DdGtIGenOiCL6niVM-3D177&d=DwMFaQ&c=slrrB7dE8n7gBJbeO0g-IQ&r=XGzUN6S7CV-sOjf7f4TPQQ&m=ZYgo-n3jro_kZjbpyVQvNmNympc-4C61WWQ_tJauGbWwf3CZ1e2EvPVeZxpfz9GE&s=PSeUPeAxclCCPF76oDAu8mAZJ7hvYz6ZFuex9OrbXPk&e=) requires authors to make all data underlying the findings described in their manuscript fully available without restriction, with rare exception. The data should be provided as part of the manuscript or its supporting information, or deposited to a public repository. For example, in addition to summary statistics, the data points behind means, medians and variance measures should be available. If there are restrictions on publicly sharing data—e.g. participant privacy or use of data from a third party—those must be specified.

Reviewer #1: Yes

Reviewer #2: Yes

4. Is the manuscript presented in an intelligible fashion and written in standard English?

PLOS Global Public Health does not copyedit accepted manuscripts, so the language in submitted articles must be clear, correct, and unambiguous. Any typographical or grammatical errors should be corrected at revision, so please note any specific errors here.

Reviewer #1: Yes

Reviewer #2: Yes

5. Review Comments to the Author

Please use the space provided to explain your answers to the questions above. You may also include additional comments for the author, including concerns about dual publication, research ethics, or publication ethics. (Please upload your review as an attachment if it exceeds 20,000 characters)

Reviewer #1: The manuscript “Countries’ Progress Towards Global Health Security Increased Health Systems Resilience During the Coronavirus Disease-19 Pandemic: A Difference-in-Difference Study of 191 Countries” leverages longitudinal data from around the world to explore the association between the Global Health Security Index (GHSI) and health systems resilience during times of crises—in this case the COVID-19 pandemic. Not only is this an incredibly important issue we need to know more about—the risk of public health emergencies continues and lessons from the last should be used for future policies—but their strategy of breaking down the index into its subcategories provides insight into areas ripe for future research.

The authors’ strategy of separating countries into treatment and control groups via one-step increments of their GHSI scores (overall and subcategories) and testing their association via a difference-in-difference model on childhood immunization coverage rates to show outcomes on health systems resilience is well thought out and posits a few very interesting concepts. One, breaking down complex indices is a viable approach to gain more specific understanding of a measure’s meaning. Two, health systems resilience’s positive association to environmental risks being more than double than the next closest subcategory (biosecurity) and having a much larger magnitude than the other components of the GHSI deserves further scrutiny by future researches and consideration by policymakers.

MAIN ISSUES
- Needs further clarification in the discussion section on how the results should be interpreted and how they align with current literature. >> Thank you for your comment, which will increase the clarity and comparability of our results. To incorporate these comments, we expanded our Discussion to better contextualize our findings. Specifically, we provided the observed percentage point declines in overall immunization coverage rates to give readers another perspective on how our results can be interpreted (Lines 381-385). In addition, we directly compared our findings on the effect sizes to the most relevant literature on this topic to provide a more comprehensive understanding of our findings in context (Lines 470-477).

- Lack of possible mechanisms for the sub-component results undercuts their importance and is in detriment of the manuscript as a whole. >> Thank you for your constructive comment, which will strengthen our sub-component findings and improve their utility for policymakers. In response, we expanded the paragraph on the sub-components (Lines 446-450) in the Discussion, positing a possible causal mechanism that may explain why governance quality is strongly correlated with health systems resilience with reference to the findings in the literature. We also expanded the first paragraph of the Discussion by adding potential causal mechanisms for why countries’ environmental scores and their emergency preparedness may have been so strongly tied to health systems resilience, again with reference to the findings in the literature (Lines 394-411), and also added a few sentences on the possible mechanisms underpinning the spike in GHS’ protectiveness in 2021 (Lines 415-425). We think this topic—possible mechanisms underpinning the sub-component results—is particularly interesting and likely has enough substance for a follow-on paper, which we would like to explore next.

ISSUES
• Line 111: Data on childhood vaccination rates should be until 2022, if not you should clarify given that your results on the outcome are until 2022. >> Thank you for catching this mistake. We have changed “2021” to “2022” (Line 120).

• Line 126: Clarify is data is missing or if the recommendation is not universal (Portugal only vaccinates BCG to children at risk, Austria it hasn’t been mandatory in a while, etc.) >> Thank you for this comment. We have added the statement “or did not report data due to differing national vaccination requirements” to address this important distinction (Lines 142-143).

• Lines 156-7: Consider briefly stating what the possible groups are (even if its explained later) since at this point it isn’t clear. >> Per your recommendation, we added in a half-sentence to provide a clear explanation of the high/low group for our main model specification (Lines 177-179).

• Line 195: The table needs at a minimum clarification of the fact that both row and column wise percentages are being presented. Consider simplifying, while the n is important to know, the total column for the Income Group and Region are not that informative. >> Thank you for this observation. To simplify the table, we deleted the column wise percentages for the World Bank Income Group and WHO Region categories (Line 265).

• Lines 260-1: Note provides insufficient information on what Control of Corruption means. >> Thank you for your feedback. We have revised the text under the table to provide a clearer explanation of what the parallel pre-trend assumption means and why Control of Corruption was therefore not included in the results (Lines 357-360).

• Lines 286-9: Unclear, do you mean that less urban area reduce COVID deaths? Less deforestation and urbanization result in a higher environmental GHS and this is protective which is reflected by lower decline in vaccination? What are the potential mechanisms? Urbanization has more health care provider availability but a higher concentration of people during the COVID-19 pandemic led to an easier spread, so what is the mechanism here? Is this in line with current findings in the literature? >>Thank you for this constructive comment and the more overarching note to elucidate the possible mechanisms behind our results. As noted above, we have expanded the discussion by providing a possible mechanism with reference to findings in the literature.

• Add if the study conforms to any relevant guidelines (CONSORT, MIAME, QUORUM, STROBE). >> We added a sentence to the end of the Methods noting that the study conformed with the STROBE guidelines and pointing readers to the STROBE checklist in the Supplementary Files. Thank you!

Reviewer #2: Thank you for the opportunity to review this interesting paper. The topic of the paper is critical as building health systems that are resilient to major shocks can prevent avoidable morbidity and mortality. In this study, the authors assess the relationship between preparedness and vaccine coverage as a proxy for resilience. They found that more prepared countries were able to improve vaccine coverage during the pandemic. While the paper is well-written, I think there should be several improvements to improve the clarity of the manuscript. The following are my comments:

Background

Lines 80 – 87: I think it is critical to define health system resilience in this section or the next. This is important point because there are many definitions of resilience in the literature and it would be important to describe resilience to the reader. It may also be helpful to briefly include what other studies have found to modulate resilience across countries and to briefly speculate why/how preparedness may also impact a countries’ resilience. >> Thank you for this comment, which will help readers better understand the scope of our paper and more easily contextualize our findings within the broader resilience literature. We derived and included a scoped definition of health systems resilience most relevant to our work from a framework recently published in The Lancet (Lines 85-87).

Lines 93: It would be helpful to briefly describe why childhood immunization coverage rates is an adequate proxy/measure of resilience. >> Thank you for this comment; we included a sentence justifying why childhood immunization coverage rates were used as a proxy measure of health systems resilience (Lines 96-99).

Lines 75-78: The following a paper may also be a helpful citation of undercounting of COVID impacts impacting analyses: [https://pubmed.ncbi.nlm.nih.gov/38879515/](https://urldefense.proofpoint.com/v2/url?u=https-3A__pubmed.ncbi.nlm.nih.gov_38879515_&d=DwQFaQ&c=slrrB7dE8n7gBJbeO0g-IQ&r=XGzUN6S7CV-sOjf7f4TPQQ&m=ZYgo-n3jro_kZjbpyVQvNmNympc-4C61WWQ_tJauGbWwf3CZ1e2EvPVeZxpfz9GE&s=rR_3wzlFE_07Qi9UYp_Fq2QGO4DBaOT6EEvaoE-6aP4&e=) >> This is an incredibly useful citation—we very much appreciate you flagging it. We think this article greatly strengthens our argument, especially given its direct use of the GHSI (Lines 78-79). Thank you!

Methods

Lines 110-111: For readers who may be unfamiliar, it would be helpful to know what age group the vaccine coverage estimates represent. For example, are the vaccine coverage estimates for children under 1 or under 15? >> Thank you for this comment; we have added an explanatory sentence to the Data paragraph in the Methods section nothing that most, but not all, vaccination estimates were for infants (children under age one), and referring readers to the WHO-UNICEF methods for a full list.

Line 112: In Figure S2, please add a caption/footnote describing what each blue line represents. I’m somewhat confused why there are two lines, and what average coverage rate means. >> We have added a new caption under the graph explaining both what each blue line represents and what we mean by average coverage rate. Thank you for this comment; the graph is now much more understandable.

Line 152-155: Can you describe how the doubly-robust method was used here? I’m not an expert in DiD methods but from my understanding, you use a doubly-robust method to protect from model misspecification when you have a propensity logistic model and outcome model. But you don’t have a propensity score model here so why use a doubly-robust method? Please justify the use of this method and the benefit of the doubly-robust property in this instance? >> Thank you for this comment; we realized that, to your point, we had not included an explanation of why we chose Sant’Anna and Zhao’s methodology, which is one of this paper’s comparative advantages. We have added a new sentence to the Statistical Analysis section, which explains this (Lines 172-175). In summary, we used this method because it allowed us to conduct a standard DiD comparison (which controls for time trends) while also implementing a regression model to adjust for potential covariates (without the propensity score model). The key benefit of the doubly-robust method in this context is that it provides an additional layer of protection against bias from **misspecification of the regression model**. If the regression model is misspecified—such as failing to include relevant covariates or misrepresenting their relationship with the outcome—the estimator can still be valid **if the DiD is correctly specified**. We were also drawn to this methodology because it allowed us to systematically test the parallel test assumption across many different model specifications.

Equation: Again, I’m inexperienced in the DiD method but it was my understanding that interaction terms between time and the intervention are needed in the outcome model. But there are no interaction terms in the equation, I assume this is needed to get the year-specific results in Table 3. >> Yes, interaction terms between time and intervention are needed in the outcome model. Our initial draft used an equation which simplified the treatment model (e.g., the interaction term) into one variable, but, as you pointed out, this introduced confusion rather than having the intended effect of making the equation more understandable. We’ve therefore rewritten Equation 1 to fully reflect the outcome model (Line 185), added in an explanation of the variables, and included another sentence about how the outcome model affects in the DiD estimator (Lines 177-183). Thank you very much for your insightful comment.

Lines 168-169: Why did the authorship team include the government indicators as a robustness check? Many of these indicators, especially the government effectiveness indicator, are already included in the GHS Index. So how is this additional analysis a robustness check? Please justify. >> Thank you for this comment. The GHS Index has an indicator for government effectiveness (6.1.1), but this indicator is derived from data sufficiently distinct from the World Bank’s governance effectiveness composite to function as a robustness check. To test this assertion, we found that the World Bank’s governance effectiveness composite has a correlation of 0.91 with GHSI 6.1.1 (government effectiveness) and a 0.74 correlation with GHSI 6.1 (political and security risk). We have added a new sentence to clarify this point in response to your comment (Lines 217-220) and we think that providing the correlations between the World Bank’s governance composite and GHSI’s sub-component on governance effectiveness makes the paper much more robust. Thank you again for helping us better underscore this distinction!


Results

Table 1: I believe this would be better communicated as a map showing what group each country belongs. Reading through the table may be too burdensome for readers. >> We replaced Table 1 with a map in the main text (Page 10), and moved Table 1 to the Supplementary Materials as Table S3.

The results section could be improved by describing trends in childhood vaccination rates and how rates were impacted during COVID. Perhaps included temporal trends of vaccinations may be helpful to improve the section. >> We added new text to the Result section’s Descriptive Analysis section which describe the temporal trends of childhood vaccination rates pre- and during the pandemic (Lines 242-247). Thank you for this comment.

Lines 201-202: It would be helpful to include an interpretation of your main effect size here rather than just providing the coefficient. >> Thank you; we have made this edit (Lines 272-274).

Many DiD studies generally present a time trend plot of the outcome by treatment group to show the effect of the intervention. I think this figure is needed to show readers the impact of preparedness on resilience. >> We observed a general trend in our paper (thanks to your and other reviewer’s comments) that we needed to do more work to better contextualize our findings in light of the literature. We therefore created a new figure (see Figure 2 on Page 12) which not only includes a time trend plot of the outcome (DiD coefficient) by treatment group, but also how immunization coverage rates changed over time, which we think helps readers understand the size and timing of the DiD effect.

Discussion

Please describe the mechanisms by which preparedness may improve resilience? Is that more prepared countries have pre-existing plans for maintaining essential health services during the pandemic? Perhaps they have a strong healthcare workforce and resources? Or perhaps they have fewer lockdowns? I think more discussion on why preparedness promotes vaccine services is needed to improve the impact of this paper. >> Thank you for this note; we agree that our original draft did not sufficiently elaborate possible mechanisms which may be driving our results. To strengthen our results, we expanded the paragraph on the sub-components (Lines 446-450) in the Discussion, positing a possible causal mechanism that may explain why governance quality is strongly correlated with health systems resilience with reference to the findings in the literature. We also expanded the first paragraph of the Discussion by adding potential causal mechanisms for why countries’ environmental scores and their emergency preparedness may have been so strongly tied to health systems resilience, again with reference to the findings in the literature (Lines 394-411). We think this topic—possible mechanisms underpinning the sub-component results—is particularly interesting and likely has enough substance for a follow-on paper, which we would like to explore next.

More discussion on why the effect size is strongest in 2021 can further improve the discussion. >> Thank you for this comment. We have expanded our discussion by postulating a number of potential mechanisms which might be driving the comparatively large effect size in 2021 with reference to the findings in the literature (Lines 415-425).

Minor: Can the authors briefly comment on the very high cutoff values for treatment effects in the analysis in the discussion? This implications for many countries because they would have substantial improvements to make. >> Thank you for this comment. We have commented on the implications of higher cutoff values for GHSI categories and sub-categories in the Discussion (Lines 386-389). Thank you again for your thoughtful and productive comments. We feel that these significantly strengthened our article.

6. PLOS authors have the option to publish the peer review history of their article ([what does this mean?](https://urldefense.proofpoint.com/v2/url?u=https-3A__c05y1x9s.r.us-2Deast-2D2.awstrack.me_L0_https-3A-252F-252Fjournals.plos.org-252Fglobalpublichealth-252Fs-252Feditorial-2Dand-2Dpeer-2Dreview-2Dprocess-2523loc-2Dpeer-2Dreview-2Dhistory_1_010f01920eee22e6-2D8cff98ad-2De74f-2D4f8d-2Dabdd-2Df7d752fe646c-2D000000_38Ixp1W5ZPJeDJG3vU-2DrIYDUZlY-3D177&d=DwMFaQ&c=slrrB7dE8n7gBJbeO0g-IQ&r=XGzUN6S7CV-sOjf7f4TPQQ&m=ZYgo-n3jro_kZjbpyVQvNmNympc-4C61WWQ_tJauGbWwf3CZ1e2EvPVeZxpfz9GE&s=OYDr7Kucqh4Ln6rgU8c-iy8IUzUtpilSkdl3-Dbo6fs&e=)). If published, this will include your full peer review and any attached files.


**Do you want your identity to be public for this peer review?** If you choose “no”, your identity will remain anonymous but your review may still be made public.


For information about this choice, including consent withdrawal, please see our [Privacy Policy](https://urldefense.proofpoint.com/v2/url?u=https-3A__c05y1x9s.r.us-2Deast-2D2.awstrack.me_L0_https-3A-252F-252Fwww.plos.org-252Fprivacy-2Dpolicy_1_010f01920eee22e6-2D8cff98ad-2De74f-2D4f8d-2Dabdd-2Df7d752fe646c-2D000000_U2-2D2MKL8ctfABMr-2DxcKPhm9hKkM-3D177&d=DwMFaQ&c=slrrB7dE8n7gBJbeO0g-IQ&r=XGzUN6S7CV-sOjf7f4TPQQ&m=ZYgo-n3jro_kZjbpyVQvNmNympc-4C61WWQ_tJauGbWwf3CZ1e2EvPVeZxpfz9GE&s=R673dBO-qxva0zQn2V56Vy8cWnaRHZRrNhCGqx4-ICw&e=).

Reviewer #1: No

Reviewer #2: No

[NOTE: If reviewer comments were submitted as an attachment file, they will be attached to this email and accessible via the submission site. Please log into your account, locate the manuscript record, and check for the action link "View Attachments". If this link does not appear, there are no attachment files.]

While revising your submission, please upload your figure files to the Preflight Analysis and Conversion Engine (PACE) digital diagnostic tool, [https://pacev2.apexcovantage.com/](https://urldefense.proofpoint.com/v2/url?u=https-3A__pacev2.apexcovantage.com_&d=DwQFaQ&c=slrrB7dE8n7gBJbeO0g-IQ&r=XGzUN6S7CV-sOjf7f4TPQQ&m=ZYgo-n3jro_kZjbpyVQvNmNympc-4C61WWQ_tJauGbWwf3CZ1e2EvPVeZxpfz9GE&s=UoqcD1mrEguF3HvIqSYK3ZdfkJKpLhGPn3gBPmovj2A&e=). PACE helps ensure that figures meet PLOS requirements. To use PACE, you must first register as a user. Registration is free. Then, login and navigate to the UPLOAD tab, where you will find detailed instructions on how to use the tool. If you encounter any issues or have any questions when using PACE, please email PLOS at [figures@plos.org](mailto:figures@plos.org). Please note that Supporting Information files do not need this step.
