# Supplementary material for: Countries’ progress towards Global Health Security (GHS) increased health systems resilience during the Coronavirus Disease-19 (COVID-19) pandemic: A difference-in-difference study of 191 countries
Source: PLOS Glob Public Health. 2025 Jan 7;5(1):e0004051. doi: 10.1371/journal.pgph.0004051 (PMC11706378; doi:10.1371/journal.pgph.0004051)
Supplement: Attachment — Submitted filename: Rebuttal letter_Nov 20_2024.docx [file pgph.0004051.s032.docx]

November 20, 2024

Dear Dr. Mukherjee,

Many thanks to you and to the reviewers for the opportunity to strengthen our manuscript titled “Countries’ Progress Towards Global Health Security (GHS) Increased Health Systems Resilience During the Coronavirus Disease-19 (COVID-19) Pandemic: A Difference-in-Difference Study of 191 Countries” (**PGPH-D-24-01417**). We especially appreciate Reviewer #2’s considerate and thoughtful comments about ways we can improve our methodology. We feel that the Methods section is much stronger now due to Reviewer 2’s comments.

Given this opportunity to review our manuscript, we additionally made minor edits throughout the draft for readability and flow. We hope that the revised manuscript meets your expectations and adequately incorporates Reviewer 2’s comments. Please do not hesitate to contact us if further clarifications are needed.

Thank you again for your support and consideration.

Sincerely,

Yesim Tozan
Associate Professor of Global Health

tozan@nyu.edu

PGPH-D-24-01417R1
Countries’ Progress Towards Global Health Security (GHS) Increased Health Systems Resilience During the Coronavirus Disease-19 (COVID-19) Pandemic: A Difference-in-Difference Study of 191 Countries
PLOS Global Public Health

Dear Dr. Tozan,

Thank you for submitting your manuscript to PLOS Global Public Health. After careful consideration, we feel that it has merit but does not fully meet PLOS Global Public Health’s publication criteria as it currently stands. Therefore, we invite you to submit a revised version of the manuscript that addresses the points raised during the review process.

Please submit your revised manuscript by Dec 18 2024 11:59PM. If you will need more time than this to complete your revisions, please reply to this message or contact the journal office at [globalpubhealth@plos.org](mailto:globalpubhealth@plos.org). When you're ready to submit your revision, log on to [https://www.editorialmanager.com/pgph/](https://urldefense.proofpoint.com/v2/url?u=https-3A__www.editorialmanager.com_pgph_&d=DwQFaQ&c=slrrB7dE8n7gBJbeO0g-IQ&r=XGzUN6S7CV-sOjf7f4TPQQ&m=NbQ_xxiOkzBtpVY8wC5UH1yY14gLNUOtZy3OzW0xU90f3WhiELq27pYob58vu56d&s=ooMlRNIA0DZ4wdtS7mfxFbNlYDXzUEMN6zDJklFgWrw&e=) and select the 'Submissions Needing Revision' folder to locate your manuscript file.

Please include the following items when submitting your revised manuscript:

- A rebuttal letter that responds to each point raised by the editor and reviewer(s). You should upload this letter as a separate file labeled 'Response to Reviewers'.
- A marked-up copy of your manuscript that highlights changes made to the original version. You should upload this as a separate file labeled 'Revised Manuscript with Track Changes'.
- An unmarked version of your revised paper without tracked changes. You should upload this as a separate file labeled 'Manuscript'.

Guidelines for resubmitting your figure files are available below the reviewer comments at the end of this letter.

We look forward to receiving your revised manuscript.

Kind regards,

Sanjana Mukherjee, Ph.D., M.Sc.
Guest Editor
PLOS Global Public Health

Journal Requirements:

**6. Review Comments to the Author**

Please use the space provided to explain your answers to the questions above. You may also include additional comments for the author, including concerns about dual publication, research ethics, or publication ethics. (Please upload your review as an attachment if it exceeds 20,000 characters)

Reviewer #1: (No Response)

Reviewer #2: I thank the authors for their diligent edits to the manuscript. The authors responded to my comments with edits to the text and additional analyses. The paper elucidates on how health system resilience can be improved in the coming years to avert preventable morbidity and mortality from shocks. I just have the following small comments:

Lines 174-177: In reviewing the Anna and Zhao (2020) citation and the corresponding code in github, it looks like the authors did combine the propensity score weighting methods and outcome model adjustment during modeling. To better reflect this, I recommend re-writing this line with the following:

“The doubly-robust DiD estimator works by combining inverse probability of treatment weighting, estimated from a propensity score model, and outcome model adjustment to appropriately adjust for covariates and time-varying factors and generate a consistent estimate of the treatment effect. The inclusion of the same covariates in the propensity score model and outcome model protects from model misspecification.”

This language better communicates what was actually done in the study and the benefits of the DR DiD approach. **Thank you for this suggestion. We have incorporated this explanation in its totality with minor edits for flow. We greatly appreciate your thoughtfulness and consideration in reviewing our Methods section, which we feel is considerably stronger thanks to your questions, comments, and edits.**

Line 244: Instead of using “significant” to describe the difference in coefficients, consider saying that the coefficient or effect size was larger compared to in 2020 or 2022. **We have made this edit throughout the results section, changing most uses of “significant” (when describing the difference in coefficients) to “greater coefficient than” or other variations with similar meaning. Thank you!**

7. PLOS authors have the option to publish the peer review history of their article ([what does this mean?](https://urldefense.proofpoint.com/v2/url?u=https-3A__track.editorialmanager.com_CL0_https-3A-252F-252Fjournals.plos.org-252Fglobalpublichealth-252Fs-252Feditorial-2Dand-2Dpeer-2Dreview-2Dprocess-2523loc-2Dpeer-2Dreview-2Dhistory_1_010f01933ea43f19-2Dd8c5c7a3-2Ddd2b-2D4b6e-2D85bb-2D7cd077a47039-2D000000_QbaGMQAmHs8HgVasZSqFneGdEM-2DWfY1vl241-5FXv1-5F7M-3D185&d=DwMFaQ&c=slrrB7dE8n7gBJbeO0g-IQ&r=XGzUN6S7CV-sOjf7f4TPQQ&m=NbQ_xxiOkzBtpVY8wC5UH1yY14gLNUOtZy3OzW0xU90f3WhiELq27pYob58vu56d&s=tcdHjUsBCKfvs0eka-rVivNQ6b4wpeZUnbyOA9RGWXM&e=)). If published, this will include your full peer review and any attached files.


**Do you want your identity to be public for this peer review?** If you choose “no”, your identity will remain anonymous but your review may still be made public.


For information about this choice, including consent withdrawal, please see our [Privacy Policy](https://urldefense.proofpoint.com/v2/url?u=https-3A__track.editorialmanager.com_CL0_https-3A-252F-252Fwww.plos.org-252Fprivacy-2Dpolicy_1_010f01933ea43f19-2Dd8c5c7a3-2Ddd2b-2D4b6e-2D85bb-2D7cd077a47039-2D000000_QYxNw48GJJBa5-5FdKg1MUt31PXPJK2cNfPe8vIe-5FWA0E-3D185&d=DwMFaQ&c=slrrB7dE8n7gBJbeO0g-IQ&r=XGzUN6S7CV-sOjf7f4TPQQ&m=NbQ_xxiOkzBtpVY8wC5UH1yY14gLNUOtZy3OzW0xU90f3WhiELq27pYob58vu56d&s=a7e9PR_hfA2piJQFrzDBFzvlNdlrpIoimKtfjXjOGFA&e=).

Reviewer #1: No

Reviewer #2: No

[NOTE: If reviewer comments were submitted as an attachment file, they will be attached to this email and accessible via the submission site. Please log into your account, locate the manuscript record, and check for the action link "View Attachments". If this link does not appear, there are no attachment files.]

While revising your submission, please upload your figure files to the Preflight Analysis and Conversion Engine (PACE) digital diagnostic tool, [https://pacev2.apexcovantage.com/](https://urldefense.proofpoint.com/v2/url?u=https-3A__pacev2.apexcovantage.com_&d=DwQFaQ&c=slrrB7dE8n7gBJbeO0g-IQ&r=XGzUN6S7CV-sOjf7f4TPQQ&m=NbQ_xxiOkzBtpVY8wC5UH1yY14gLNUOtZy3OzW0xU90f3WhiELq27pYob58vu56d&s=kgKMzhXCzzM_TwOXoYzz0CxvYWmq5E7k74zwDdbtD58&e=). PACE helps ensure that figures meet PLOS requirements. To use PACE, you must first register as a user. Registration is free. Then, login and navigate to the UPLOAD tab, where you will find detailed instructions on how to use the tool. If you encounter any issues or have any questions when using PACE, please email PLOS at [figures@plos.org](mailto:figures@plos.org). Please note that Supporting Information files do not need this step.
